# Exploring the influence of urban art interventions on attraction and wellbeing: an empirical field experiment

**DOI:** 10.3389/fpsyg.2024.1409086

**Published:** 2024-12-05

**Authors:** Margot Dehove, Jan Mikuni, Nikita Podolin, Martin Karl Moser, Bernd Resch, Linda Doerrzapf, Pia Marlena Boehm, Katharina Prager, Helmut Leder, Elisabeth Oberzaucher

**Affiliations:** ^1^Vienna Cognitive Science Hub, University of Vienna, Vienna, Austria; ^2^Department of Cognition, Emotion, and Methods in Psychology, Faculty of Psychology, University of Vienna, Vienna, Austria; ^3^Department of Geoinformatics, University of Salzburg, Salzburg, Austria; ^4^Center for Geographic Analysis, Harvard University, Cambridge, MA, United States; ^5^Geosocial Artificial Intelligence, IT:U Interdisciplinary Transformation University Austria, Linz, Austria; ^6^Research Unit Transportation System Planning, Institute of Spatial Planning, Vienna University of Technology, Vienna, Austria; ^7^Faculty of Life Sciences, University of Vienna, Vienna, Austria; ^8^Department of Behavioural and Cognitive Biology, Faculty of Life Sciences, University of Vienna, Vienna, Austria

**Keywords:** urban intervention art, street, mobile eye-tracking, wellbeing, field experiment, urban design, aesthetic evaluation

## Abstract

While cities are attractive places, brimming with opportunities and possibilities for their inhabitants, they have also been found to have negative consequences, especially on physical and mental health. In a world of ever-growing urban populations, it is important to understand how to make cities healthier and more pleasant places to live. In the present study, we investigated the impact of art as an urban intervention and compared it to the well-known effects of greenery (i.e., plants and vegetation) in an identically framed intervention. Specifically, we looked at how people engage with a Graetzloase (a type of parklet) and its embedding urban environment in terms of visual and spatial attraction as well as wellbeing. The Graetzloase displayed either abstract art or greenery and was placed on two distinct streets that, among other elements, also contained art and greenery. Our field study captured the ongoing experiences during people’s exploration of the urban environment by employing mobile eye-trackers and physiological devices. While our findings demonstrated a certain level of visual and spatial attraction towards the Graetzloases, it was not as pronounced as initially anticipated. Nevertheless, our analyses still inform on *What* decorating element should be placed in a Graetzloase, as well as *Where* to implement the Graetzloase. Our results suggest that artistic elements are more visually attractive (i.e., they were looked at for longer times) than the greenery, and that both visual and spatial attraction towards the Graetzloases are greatly impacted by the street context. We found that the Art Graetzloase when displayed in a wide street containing greenery elements, is visually more present in the participant’s visual field than all the other experimental combinations. The more precise analyses of the participant viewing behavior confirm this trend. Regarding wellbeing, we found no evidence for the impact of street context or the types of decorations in the Graetzloases. Our results establish an initial empirical foundation for the design and placement of not only future parklets but also urban art interventions in general.

## 1 Introduction

Cities are attractive places to live in. Paradoxically, they are also brimming with various threats for their inhabitants, including a risk of mental health issues, such as depression and psychosis (Sundquist et al., 2004). With the rapid growth of urban populations (United Nations Department of Economic and Social Affairs, 2018), understanding the impact of cities on their inhabitants as well as developing ways to improve their lives and wellbeing becomes more critical.

One well-documented urban element that has a positive impact on human wellbeing is greenery (i.e., elements such as sidewalk plants or urban gardens). The interaction with urban greenery can improve wellbeing ranging from physical and psychological to social and environmental wellbeing of the urban populations [see Jabbar et al. (2022) for a review]. Various (evolutionary) theories and hypotheses have been suggested to explain why greenery has a beneficial effect on humans. Wilson’s (1984) Biophilia Hypothesis postulates that the attraction to nature that humans routinely experience is innate and universal. Ulrich proposed that safe, non-demanding, moderately complex, open, natural environments promote physiological and emotional stress recovery [see the Stress Reduction Theory (SRT), Ulrich et al., 1991; Ulrich, 2023; Subiza-Pérez et al., 2021]. Kaplan and Kaplan argued that natural settings, by offering “soft fascination,” can engage bottom-up attention (externally driven and effortless) and thus letting the mechanism behind the top-down attention (internally driven, voluntary, and effortful) an opportunity to rest and restore [see the Attention Restoration Theory, ART; Kaplan and Kaplan (1989) and Sullivan and Kaplan (2023)]. Recently, Meidenbauer et al., 2020, introduced an alternative mechanism for the positive effect of greenery on wellbeing. They propose that the positive effect is driven not by an inherent ability of green spaces to enhance wellbeing, but by a preference for certain environments. As natural environments are generally preferred, they tend to have the greatest positive impact on wellbeing.

In the field concerned with wellbeing, there is a growing discussion about the potential of another element to improve individuals’ wellbeing. This element is art (Clift et al., 2021; Fancourt and Finn, 2019). Such effects of art were mainly studied in the context of museums (Clow and Fredhoi, 2006; Mastandrea et al., 2018). Recently, more studies have started focusing on art in urban environments (see Mitschke et al., 2017 for an example of a field study or Zebracki, 2013 for a survey of public perception). However, studies directly demonstrated the potential of art in urban environment in relation to wellbeing are yet scarce. We consider the incorporation of art within urban environments as a good candidate for enhancing wellbeing considering aesthetic experiences significantly influence one’s state of wellbeing (Mastandrea et al., 2019). Trupp et al. (2024) explored the mechanisms behind art viewing’s positive impact on wellbeing, identifying five key themes: affective processes (emotions, stress regulation), cognitive processes (sensory stimulation, learning), social processes (shared experiences), self-transformation (self-reflection), and resilience (coping with challenges). Notably, affective processes and resilience overlap with mechanisms found in theories and hypotheses on greenery-induced wellbeing (e.g., the preference of Meidenbauer et al., 2020 and the ART of Kaplan and Kaplan). Given that art is recognized as a prototypical entity capable of promoting aesthetic experiences (Leder et al., 2004), a rationale exists for investigating its impact within urban environments to confirm its potential positive wellbeing effects.

Having identified art and greenery as two potent elements influencing wellbeing, the subsequent step was to determine an optimal strategy for their integration within an urban context. We suggested parklets to promote art and greenery in public spaces. Parklets are small structures—occupying a parking spot—that extend urban public spaces and provide additional amenities for residents (Alumbaugh et al., 2013). For example, they provide the possibility to sit and present visual elements like plants, etc. Various cities (such as London, San Francisco, and Melbourne, respective citations: London Living Streets, 2018; Alumbaugh et al., 2013; Stevens et al., 2023) have adopted the concept of parklet as a strategy for participatory design of urban space. In 2015, the city government of Vienna launched its own parklet programme, called *Graetzloase*. The Austrian word Graetzloase can be decomposed into “Graetzl,” meaning “neighborhood” and “Oase” meaning “oasis.” All Viennese citizens can apply and propose ideas for their own Graetzloase and thus have their small oasis in the city. As a result, the city of Vienna hoped to promote the creation of shared spaces, where inhabitants can mingle and participate in the shaping of the public space (Brait and Hammer, 2017). Compared to pre-existing structures of the urban environment (e.g., buildings, pavement, roads), parklets have advantages for their realization, as they can be temporary, flexible, and easy to implement in urban environments, such as squares and streets. However, despite their flexibility and potential to expand urban public spaces, empirical studies on parklets in terms of how they would be used and if they could contribute to urban wellbeing have been scarce [see Stevens et al. (2023) for an example where the utility and place-making aspects of the Parklet were investigated but wellbeing outcomes were not directly measured].

Art and greenery were not only chosen for their wellbeing potential, but also because when the greenery is framed in the Graetzloase, it acts as an active control of the art (Boot et al., 2013). An active control, as opposed to the traditional control groups that have “no-contact” or “no-treatment,” is a group that receives a treatment that is different from the experimental intervention, but which is thought to be an active and credible comparison. Implementing an active control group (the greenery in the Graetzloase for the current experiment) that matches the expectation of the tested condition (the art in the Graetzloase) rather than a “no-contact” control (the Graetzloase alone, or just the street without any Graetzloase), controls for non-specific factors (i.e., participant’s motivation and expectations) that could influence our outcomes.

Moreover, with the integration of art and greenery within the Graetzloase, it is essential to recognize that these elements are situated within a broader urban context, specifically the street environment in which the Graetzloases were located. Notable works on urban environment debated that the structure of said context (e.g., where and how to design public spaces—both small elements such as our Graetzloase and more global elements such as streets) is key to promoting urban life quality (Gehl, 2010; Lynch, 1960). The significance of the structure of the public spaces is underscored by high urban population density, accompanied by the rapid urbanization. For urban residents with limited home spaces, public spaces offer the opportunity for socialising, physical activity, and leisure [see Von Szombathely et al. (2017) for a model discussing the interplay between the urban structure—or morphology—and urban health]. Moreover, a well-known principle of architecture resides in the fact that the shape and structure of a building (here extended to the urban environment) are intimately linked to its function [see Pais (2024) for a development of Louis Sullivan’s idea of “Form follows Function”]. Extending this principle, one can say that the structure of the urban environment (form) can influence its usability (function), and hence possibly people’s behavior (Dietrich and Kengyel, 2016). With the urban structure and context playing such an important role in usability and wellbeing, we decided to place the Graetzloase on two streets which had different structures. One street was located in the city center, with narrow sidewalks which put people quite close to the traffic, murals on one of the shop façade and very few greenery. The other one was in the suburbs and offered a comfortable width of sidewalks and vegetation (see Table 1 for a precise description of the two streets).

**Table 1:** Characteristics of the two testing streets [table adapted from Mikuni et al., 2024 and refined with Hillnhütter, 2021].

	Art-Street (testing area side)	Green-Street (testing area side)	Art-Street (the opposite side of the testing area)	Green-Street (the opposite side of the testing area)
Characteristics				
Car restrictions and traffic	Bike-friendly, car-dominated, occupied parking spots, one-way traffic with two lines	Bike-friendly, traffic calmed, the parking spaces were not so occupied, two-way traffic with two lines	Same as the opposite side	Same as the opposite side
Shop and services	Five shops - Bed/Linen - Elevator and Alarm systems - Arts and crafts - Motorbike (closed?) - Beauty shop Pub × 1	Four shops - Mobile - Tobacco- Finance - Bank	Two shops - Agricultural products - Vintage shop for furniture (in the parking lot space) Kinder Garden/Nursery school × 1 Restaurant × 1 Office × 1: Accounting	Two shops - Supermarket - Stationer’s shopRestaurant × 1
Enclosure and accessibility (physical/visual)	Narrow streets, no squares Narrow sidewalks, unfriendly for disabled or blind people	Comfortable width streets, small size squares/gardening areas Relatively empty and wide spaces	Same as the opposite side	Same as the opposite side
Height of the buildings	- 4 floors × 3 - 6 floors × 1 - 2 floors × 1	- 6 floors × 2	- 4 floors × 4	- 6 floors × 1 - 7 floors × 1
Social activities	Walking, no Stationer’s shop activities apart from the Graetzloase	Walking, some Stationer’s shop activities apart from the Graetzloase (metallic chairs to sit but were in a restricted area).	Walking, no Stationer’s shop activities	Walking, no Stationer’s shop activities
Territorial markers/structures indicating territorial borders Other amenities	Cross-road marks at the beginning and end of the street block (out of the testing area) No benches, no playgrounds, some bike stands	Cross-road marks at the beginning of the street block (in the testing area), traffic signs on the street, bus stop (out of the testing area) Bins and metallic chairs (the latter in restricted area)	Same as the opposite side	Same as the opposite side except for no traffic signs on the street and no bus stop
Greenery	No trees, no water, pub terrasse had small flowers in pots No urban gardening	There are several small size trees on the street, no water Two urban gardening areas	No plants, no trees, no water, or any natural elements No urban gardening	No plants, no trees, no water, or any natural elements No urban gardening
Number of buildings Edges, facades Streetscape appearance	Five buildings (four buildings + one artistic building) Burggasse 98’s facade has a mural, variation in the types (shape and colors) of facades, faces with strong identity, old buildings, clean, well maintained	Two building (apartments, both having shops on the first floor) No decorative buildings in this area; all facades look the same (monotonous); modern buildings, clean, well maintained, somewhat boring	Four buildings (two of them are more visually salient: pink wall, big glass windows, modern structure)	Two buildings (apartments, both having shops on the first floor)

**Figure 1:**
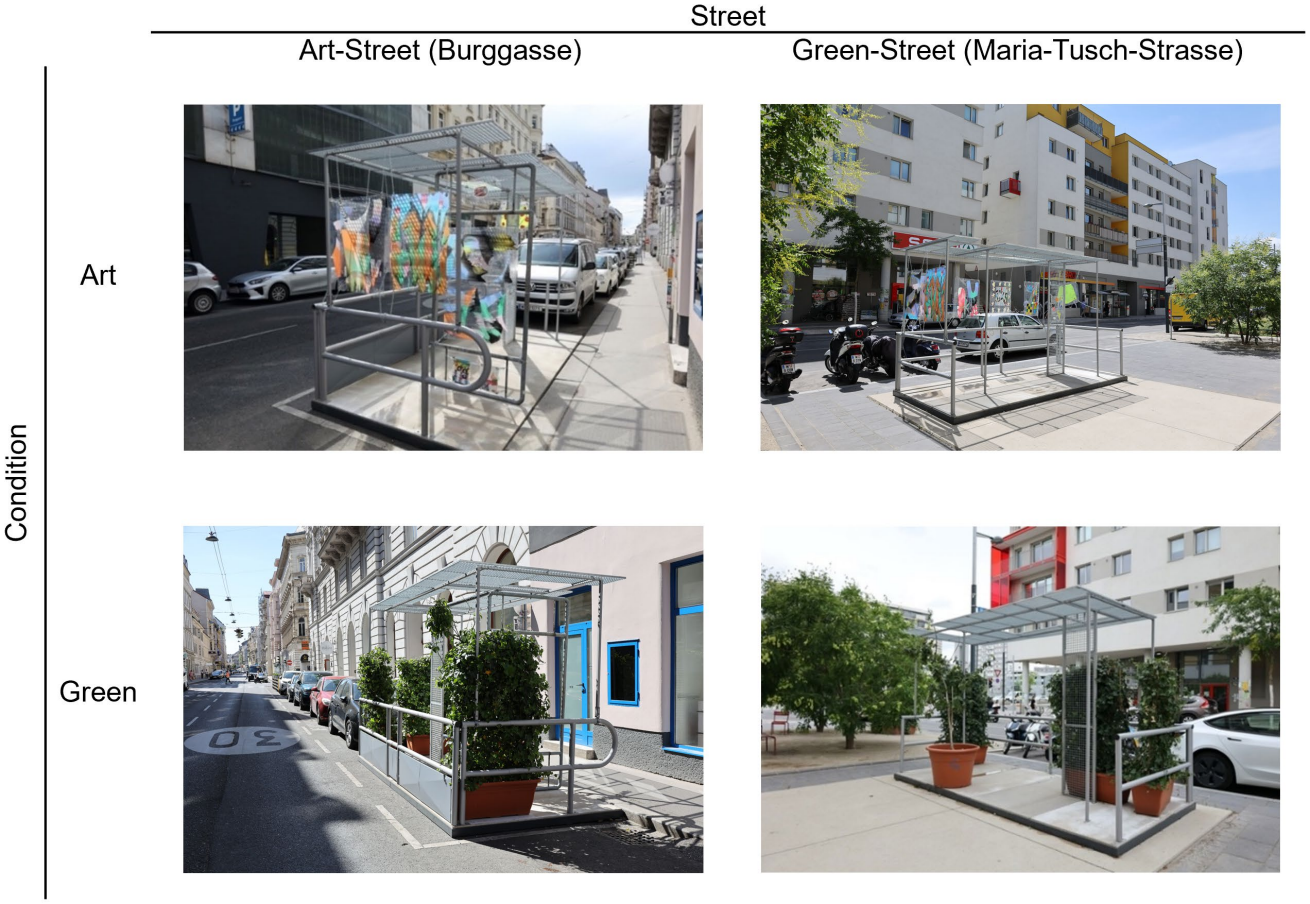
The four different experimental scenarios.

This study is motivated by the potential of the Graetzloase parklets and artworks to promote the usage of urban public spaces and improve urban wellbeing. The aims of the present study were to see: (1) if Art^1^ in the urban environment (i.e., framed in a Parklet and in a street) can impact wellbeing, (2) if people would be attracted to this new type of urban element on the streets, especially if equipped with Art. While some research has been conducted on urban art and wellbeing, direct comparisons are limited, (3) hence why we also explored the different relationships between attraction, wellbeing, and subjective experiences. To investigate these aims, we conducted a field experiment, where we implemented two Graetzloases on two distinctively different streets in Vienna, Austria. The two Graetzloases were either decorated with artistic (i.e., laminated abstract art prints; see OSF folder called “Pictures_UrbanEnvironment_And_Parklets” for detailed pictures) or with green elements (i.e., four potted plants, see OSF folder called “Pictures_UrbanEnvironment_And_Parklets” for detailed pictures). Figure 1 provides an overview of the Graetzloases. The following lines further develop our working hypotheses for each aim.

### 1.1 Aim 1: art, Graetzloase, urban environment, and wellbeing

We first wanted to note that wellbeing is a multifaceted term that is complex to define (Dodge et al., 2012). Nevertheless, according to the World Health Organisation, a state of mental wellbeing is a state that “enables people to cope with the stresses of life, realize their abilities, learn well and work well, and contribute to their community” (World Health Organization, 2022). In the context of this study, we focused on measuring acute stress which can be caused by external stimuli such as art and greenery and related to negative wellbeing (i.e., a decrease in wellbeing). More precisely, we used physiological signals representing reactions of the human body in accordance with an (external) stimulus. Primarily, we use Electrodermal Activity (EDA), the skin conductance of the human body, which provides a biomarker that has frequently been related to acute stress. It represents the sympathetic nervous system (SNS) reaction—the system that is activated when one faces stressful situations (Kyriakou et al., 2019).

We apply a methodology proposed by Moser et al. (2023), where the raw sensor measurements were processed by filtering the EDA signal with a bandpass filter to remove noisy measurements and separate the body’s stress response, represented as a prompt rise in a person’s skin conductance, from increased skin conductivity that is caused by physical activity. Next, the Moments of Stress (MOS) algorithm to the filtered measurements was applied, where we set the first 5 min as the baseline, the state where the test subjects are non-stressed. Based on deviations from this baseline, rules serving as indicators of stress are applied and added to a continuous MOS score. At the end, we apply a binary threshold to the MOS score to arrive at a binary indicator for stress, 1 being stressed, and 0 being not stressed. More details on the stress algorithm and the pre-processing steps involved can be read in Moser et al. (2023). Relating the binary stress variable to the concept of wellbeing, a MOS would be a negative wellbeing factor (i.e., more MOS indicates a decrease in wellbeing).

Since the MOS methodology has been applied in laboratory settings and field studies involving cyclists and pedestrians (Kyriakou et al., 2019), we aim to test whether urban artistic or green stimuli have the potential to elicit a (non)-negative stress response that can be derived from the physiological reaction of an individual’s body. Nevertheless, we did not have any specific hypothesis regarding whether the Art Graetzloases would have a greater impact on the MOS than the Green one. However, we thought that the environment where the experiment took place could have an impact on wellbeing as gauged by MOS. Indeed, as previously mentioned, the two streets where the Graetzloases were placed were very different in nature (i.e., one street was in the busy city center and the other one in the calmer suburbs). Physiological and emotional stress recovery are promoted by safe, non-demanding, moderately complex, open, natural environments (see SRT). We thus hypothesized a strong effect of the context: both Green and Art Graetzloases placed in the narrow and deprived of natural elements street would evoke more MOS compared to the two Graetzloases placed in the greener and wider street.

### 1.2 Aim 2: art, Graetzloase, urban environment, and attraction

Due to the novel aspect of the concept of parklet equipped with art on a street, we wanted to see how people would behave around it (e.g., would they use the Graetzloase?). In the context of this study, we focused on attraction towards the Graetzloase to evaluate behavior (i.e., the potential of the Graetzloase to be used). Attraction was measured in two ways: visual attraction and spatial attraction. We defined visual attraction as the time the participant dwelled at the Graetzloase (Bojko, 2013), and spatial attraction as the time the participant spent inside the Graetzloase.

As Graetzloases are salient, new, and interesting objects in the urban environment we expected that people would notice and be visually and spatially attracted towards Art and Green. As previously mentioned, human attraction to greenery has been explained in many ways (Biophilia Hypothesis, STR, ART, aesthetic appeal). Regarding the attraction for art, past literature highlights that art in general is an attractive object as it provides affective (emotions, pleasure, etc.) and cognitive (challenges your views, etc.) experiences (e.g., Leder et al., 2004). Now for the specific case of why art could be attractive in an urban environment, it is likely that as art is classically displayed in a museum, seeing art on the street could be intriguing to the city dwellers.

Nevertheless, we primarily thought that the context the Graetzloases are embedded in would play a significant role in how people behave towards the Graetzloases. When looking at urban art and attraction, past related literature report context effects in either different contexts (e.g., Gartus et al., 2015 measured visual behavior but contrasted modern vs. graffiti artworks in either a museum or a street context) or with different measures (e.g., Motoyama and Hanyu, 2014 used an urban context but measured visual properties and affective appraisals) than the ones we used for the present study. More recently, Hillnhütter (2021) investigated how stimulating urban walking environments are. One of her conclusions is that visual variation of the walking environments appeals to pedestrians’ senses, especially at a short distance from the pedestrian. Hence, we argue that the context in which the Art and Green Graetzloases were placed can influence people’s attraction towards the Graetzloases, especially when the two streets in which the Graetzloases were implemented had very different characteristics. By having: both green and artistic elements in the Graetzloases, as well as both art and greenery within the street in the Graetzloases are embedded, allows the study of the impact of contrasting vs. continuous design. Indeed, if the Green Graetzloase is placed in a street which also has a lot of green elements, one could say that the design is continuous (the same goes for the Art Graetzloase in the street with murals). On the other hand, the couples Green-Street/Art-Graetzloase and Art-Street/Green-Graetzloase are considered to provide more contrasted compositions (i.e., contrast between the Graetzloase elements and context). We hypothesized that the contrast effect visually and spatially attracts the participants more towards the Graetzloases as the contrast challenges people’s expectations of their surroundings.

### 1.3 Aim 3: relationships between attraction, wellbeing, and subjective experiences

Past studies in art psychology have repeatedly documented that objects that have high aesthetic values (e.g., preference, beauty), regardless of the types of stimuli, receive longer looks [see Holmes and Zanker (2012) for diverse objects; see Shimojo et al. (2003), Leder et al. (2010, 2016) for faces; see Goller et al. (2019), Mitrovic et al. (2020), Shimojo et al. (2003) and Mikuni et al. (2022) for art and abstract designs]. This conclusion seems to be applicable to urban environments (Mitschke et al., 2017; de la Fuente Suárez, 2020). Past literature investigating cities also highlights that exposure to aesthetically pleasing environments can enhance individuals’ wellbeing (Galindo and Hidalgo, 2005; Galindo and Rodriguez, 2000). To check if these established findings are applicable to the Graetzloases, we decided to investigate the correlations between visual/spatial attraction, wellbeing state, and participant’s subjective evaluations. The subjective evaluations were about participants’ general experience, the urban environment, and the Graetzloase. The evaluations of the general experience gauged the participants’ aesthetic experience as well as their perception of how long the time spent in the street was. The evaluation of the urban environment was done with a questionnaire about the restorative potential of the said urban environment (Hartig et al., 1997). And finally, the Graetzloases themselves were also evaluated on their aesthetics. Based on the results of the past studies, we predicted that some correlations would be statistically significant. When people have an aesthetically pleasing experience of the Graetzloase, they might look for longer times (visual attraction) at the Graetzloase. Similarly, spending a longer time inside the Graetzloase (spatial attraction) equipped with Art and Green might be a sign that the participant has a positive aesthetic experience of the Graetzloase. Finally, positive aesthetic evaluations of the general experience and of the Graetzloase might be linked to a positive impact on the wellbeing that is felt during the experience of urban public space.

## 2 Materials and methods

### 2.1 Participants

A total of 130 participants took part in the study (73% female, 25% male, 2% other gender, *M*_age_ = 24.96, *SD*_age_ = 8.09, range: 18–73 years).^2^ To allow for unforeseen issues that could have happened during the experiment (e.g., bad weather, missed follow-up visits) or at the level of the data analysis (e.g., data loss, bad data quality), it was decided to collect as much as possible data. A total of 130 people was the maximum number we could possibly collect in the conditions of the experiment (i.e., dependent on the weather and on people’s ability to come back for their second visit). Note that out of the 130 people who took part in the experiment, not all could be included in the data analysis (see “2.6 Pre-processing and data cleaning” for more information). *A posteriori* power analysis is reported in the Results section (see “3.2 Planned Analyses” for more information). As physiological responses were measured, participants with any major health problems (e.g., heart-related conditions, neurological or physiological impairments) were excluded at the recruitment phase. All participants had normal or corrected-to-normal vision. In the latter case, participants were asked to wear their contact lenses, and not their glasses, so that they could wear the eye-tracking devices. Finally, participants were asked not to wear any eye makeup. Participants who could speak English or German were eligible to participate. Participants received 40€ after completing the second visit. This experiment was conducted in agreement with the Declaration of Helsinki’s ethical standards and with the ethical regulations in place at the University of Vienna.

### 2.2 Testing areas: streets and Graetzloases

The Graetzloases and their decorative elements (Art and Green) were the fruit of a close collaboration with a group of local artists (Burggasse 98 collective and the Studio Tinus). The base structure of Graetzloases was composed of a ground-level platform paired with a pergola-type structure which was made of upcycled metallic elements (e.g., former bike stands). The entire interventions were 4.5 m long, 2.10 m wide, and 2.53 m high. The artworks consisted of laminated abstract pieces that looked like stained glass. They were attached to the pergola structure, giving a window-like impression to the Graetzloases. The greenery consisted of four large potted green plants. Graetzloases were matched in the amount of art and greenery. Additional pictures of the artworks and greenery are available on the OSF (see “Pictures_UrbanEnvironment_And_Parklets” component).

The effort described here to have the Art and Green conditions to match were done to frame Green as the active control of the Art. We also further argue that the Green condition was a good active control (matched for motivation and expectation) for the Art based on two types of evidence: (1) past literature and (2) the analysis of some data about the subjective experience from Mikuni et al. (2024) (the study that shared the same data collection as the present one). In the past literature, it has been shown that both exposure to art and nature (especially green spaces) positively influence wellbeing, including mental health and happiness. Art, particularly through aesthetic experiences, can improve mood, reduce stress, and enhance cognitive and emotional states, ultimately promoting wellbeing (Mastandrea et al., 2019). Art is also a particularly attractive type of stimulus (e.g., Leder et al., 2004). Similarly, spending time in nature, especially green environments, has been linked to increased happiness, reduced stress, and better mental health outcomes. In addition, as previously mentioned, greenery is also very attractive (see Biophilia Hypothesis, STR, ART, aesthetic appeal). Without entering the details of the mechanisms behind art and greenery impact on wellbeing and attraction, one cannot help to notice that they are effective tools for enhancing overall wellbeing, and hence making Green a good control for Art when testing the effect of art on wellbeing and attraction. The subjective evaluation of the intervention and the walk reported by Mikuni et al. (2024) can also further validate the fact that, in the case of this specific study setup, greenery was a good active control for the art. Indeed, all the mean values for the affective (beauty, liking, enjoyment) and cognitive (meaningful, refection) aspects are close together (see Table 6 for the Intervention and Table 7 for the general experience of the walk), indicating that the four different walks offered a similar experience to the participants.

Graetzloases, decorated either with artistic or green elements, were built on two urban streets in Vienna: Burggasse in the district of Neubau and Maria-Tusch-Strasse in the district of Seestadt. Pictures of the Graetzloases in the two different streets are shown in Figure 1.

**Figure 2:**
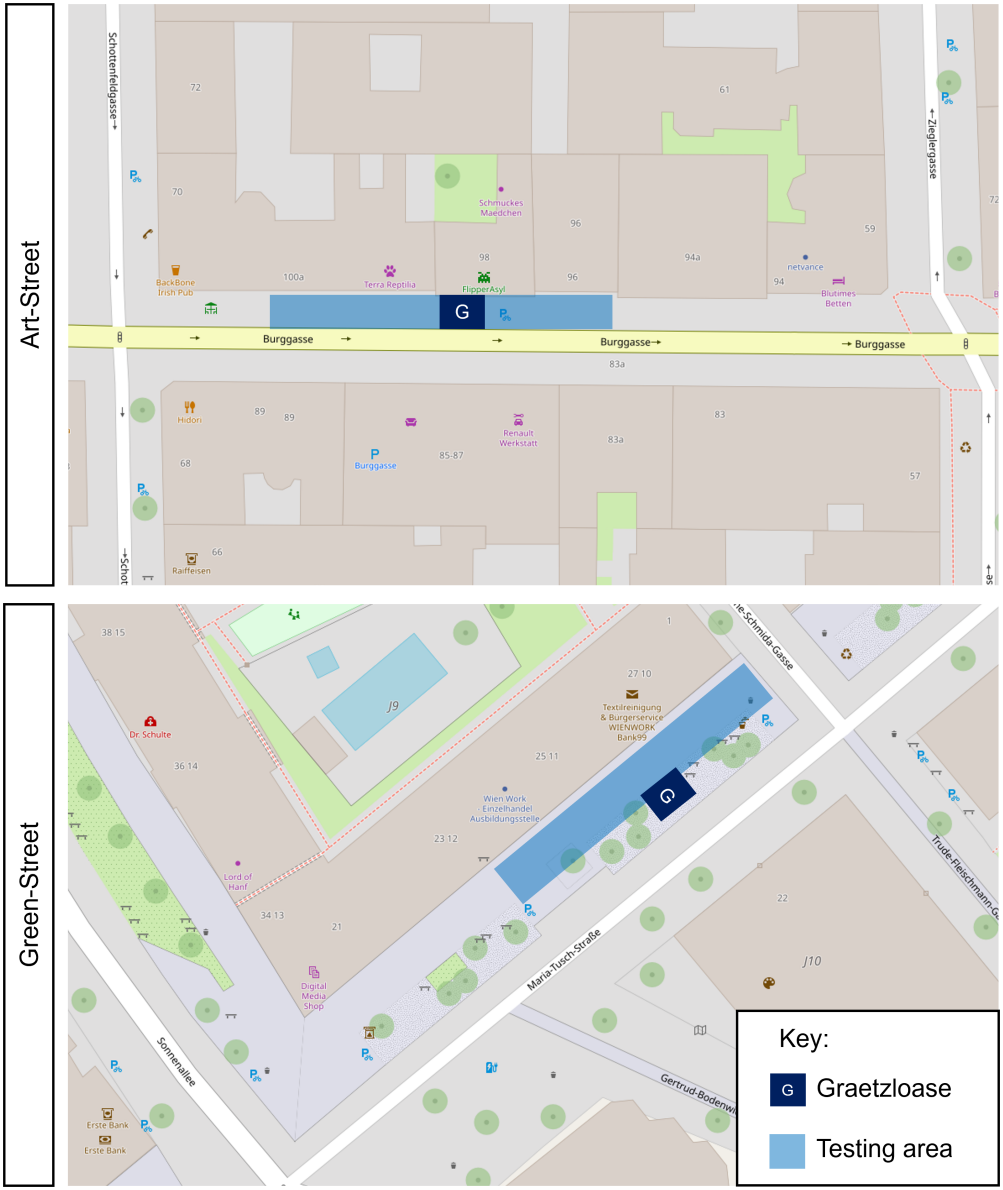
Position of the Graetzloases on the two streets. Note. Images obtained from the OpenStreetMap website (OpenStreetMap, 2024). Scale 1:720. Image downloaded with the Standard layer at 2663 x 1386.

Burggasse is a street with narrow sidewalks, deprived of natural elements, and with contemporary murals. This street is hereafter referred to as the Art-Street. Maria-Tusch-Strasse is a street which has wide sidewalks containing natural elements. This street is hereafter referred to as Green-Street. Table 1 provides a full description of the streets’ characteristics. A map of the two streets indicting the position of the Graetzloases is available in Figure 2. Moreover, complementary pictures of the street environment are provided on the OSF (see “Pictures_UrbanEnvironment_And_Parklets” component).

### 2.3 Experimental design

A mixed design was adapted with two factors. *Street* (Art-Street vs. Green-Street) was the between-subject factor, and *Condition* (Art Graetzloase vs. Green Graetzloase) was the within-subject factor. Participants were randomly assigned to either one of the streets (Art-Street or Green-Street). They visited the same street twice to be exposed to both the Art Graetzloase and the Green Graetzloase. The order of presentation of the condition was counterbalanced. To avoid carryover effect from the previous visit (i.e., a memory effect for the second visit), there were at least 12 days in between the two visits. On average, participants came back after 21 days (*SD* = 5.10), and the longest it took for one participant to come back is 36 days.^3^

### 2.4 Material

#### 2.4.1 Eye-tracking devices

Participants’ visual and spatial attractions were measured with mobile eye-tracking devices. Two different eye-trackers were used: the Tobii Pro Glasses 3 (Tobii Technology, Stockholm, Sweden) and the Pupil Invisible Glasses (Pupil Labs, Berlin, Germany). The Tobii Pro Glasses 3 were connected via wire to a recording unit that contained an SD card, where the data were stored. The glasses were controlled via the Tobii Pro Glasses 3 controller application (Tobii Technology, 2020), which was installed on an Android smartphone. Pupil Invisible Glasses were directly connected to the Android smartphone running the Pupil Invisible Companion app (Pupil Labs, 2019). The main characteristics of the eye-trackers are reported in Supplementary Table S1 in the supplementary material.

#### 2.4.2 Wellbeing device

Electrodermal activity (EDA) was recorded via a non-intrusive wristband, the Empatica E4 (Empatica, Boston, United States). The portable nature of the Empatica E4 enables the real-time measurements of the physiological signal. The wristband was wirelessly connected to a mobile smartphone (Samsung Galaxy) via Bluetooth. The recorded data were saved via on the eDiary app (Petutschnig et al., 2022), which was installed on the smartphone. The EDA was measured at a frequency of 4 Hz, with a resolution of 900 pico Siemens per digit and with a range of 0.01–100 μS (Empatica SRL, 2014). Next to EDA, the E4 wristband also measured blood volume pulse (BVP), inter-beat interval (IBI), as well as skin temperature (ST). However, they were not considered for the present analysis as they were not used to compute the variable of interest [i.e., the moments of stress (MOS)], which is derived from the EDA signal based on a rule-based algorithm proposed by Moser et al. (2023).

#### 2.4.3 Subjective evaluations of the general experience, urban environment, and Graetzloase

All subjective evaluations were collected on the LabVanced online platform (Scicovery GmbH, Paderborn, Germany; Finger et al., 2016). Participants answered the subjective evaluations on their personal phones by scanning a QRcode linked to the platform. Participants’ demographic information (i.e., age and gender) were collected at the beginning. To better understand participants’ experience of the testing area, we collected participants’ evaluation of their general experience, the surrounding urban environment as well as on the Graetzloase.

Participants’ general experience was evaluated by three statements with seven-point Likert scales (1 = not at all to 7 = very much so): enjoyment, meaningfulness, and the duration of the experiment. The surrounding urban environment was evaluated with the Perceived Restorativeness Scale (PRS; Hartig et al., 1997), assessing how restorative the participant found the testing location. The PRS is a questionnaire comprising 26 items (0 = not at all to 6 = completely) which can be grouped into four sub-scales: Being Away, Fascination, Coherence, and Compatibility. An average value of the answers to the 26 items was computed to obtain a global PRS score (Hernandez and Hidalgo, 2005; Purcell et al., 2001). Examples of items comprise: “Being here is an escape experience,” “There is much to explore and discover here,” “It is a confusing place,” “I have a sense that I belong here.” The Graetzloase was also evaluated on both affective (liking and beauty) and cognitive (meaningfulness and reflection) aspects of aesthetics (Leder et al., 2004), using seven-point Likert scales (1 = not at all to 7 = very much so).

### 2.5 Procedure

**Figure 3:**
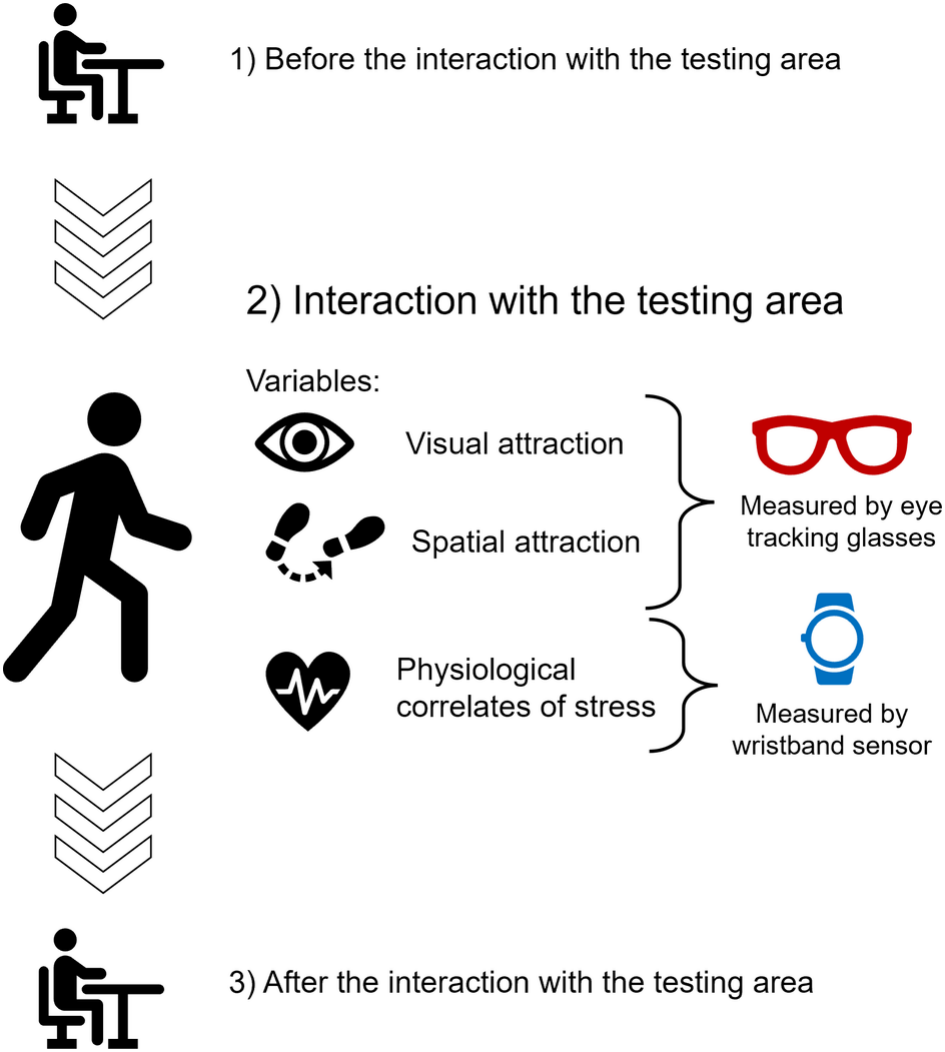
Visual summary of the experimental procedure.

Data collection took place between 2 May and 28 July 2022, on weekdays from 9 a.m. to 3 p.m., under favorable weather conditions. Participants were recruited via two online recruitment platforms. The location was unknown to the participants at the time of their sign-up to avoid a skewed choice for one location or the other. The procedure for one session had three main steps. A detailed description is provided below, and a visual summary is available in Figure 3.

1. *Before the interaction with the testing area:* Upon arrival of the first visit, information about the experimental procedure as well as the safety of the equipped devices were provided to the participants. If participants did not have any questions, they signed the consent form. The eye-tracking glasses were installed on the participant’s faces. As far as possible, we tried to give the same pair of glasses to the participants for both their visits. If they had long hair, they were asked to tie it up. To reduce the impact of bright light directly on the eye-tracking glasses, which can impair the data quality, participants wore a white cap. The participant also wore the E4 wristband to record EDA. As both eye-tracking and wellbeing devices were linked to mobile phones, participants had a small bag to carry them. The calibration of the glasses was then performed. For the Tobii glasses, a one-point type of calibration procedure was done with a fixation target drawn on a calibration card. Participants had to look at the center of the target, which was placed approximately 1 m away from the participant at the level of their eyesight. The Tobii Pro Glasses 3 controller application provided feedback if the calibration was successful or not. Due to its gaze estimation technique, the Pupil Labs Invisible glasses did not need any specific calibration step (see Tonsen et al., 2020). To control for the calibration, we further asked participants to look at four chosen landmarks at the experimental base to visually check the accuracy of the gaze detection. If the gaze reported by the app matched the landmark, the calibration was judged as correct. Participants then sat down for 5 min and answered a series of questionnaires evaluating their subjective wellbeing, and their physiological activity was recorded for 5 min. Note that we will not elaborate on the results of these questionnaires and measurement of physiological activity performed before the interaction with the testing area, as they are not the focus of the present study. These analyses are reported in Mikuni et al. (2024).
2. *Interaction with the testing area:* The testing areas were a 30-m long portion of the street where the Graetzloases were implemented (see Figure 2). It was delimited by two street landmarks (Art-Street: from the end of the Bed/Linen shop to the Beauty shop sign; Green-Street: from the entrance of the Tobacco shop to the corner of the Bank). Participants were free to interact within the testing area containing the Graetzloase for 5 min. More precisely, participants received the following instructions upon starting the 5-min interaction: “You are going to interact with the urban space for 5 min. When 5 min are over, we [one of the researchers] are going to talk to you, so you do not have to worry about the time. The testing area will be from here to there [depended on the testing location]. Please only explore this area during the testing. During the testing, do not drink, eat, or smoke. Drinks and snacks will be available for you at the end of the experiment. Do not chew any gum or look at your personal phone while walking. When you explore the testing area, please do not jump and run; just take a walk as you would normally do. Please also do not go into the shops. Other than that, there are no restrictions.” Experimenter made sure that the participants were aware of the limits of the testing area. Note that the experimenter oversaw the monitoring of the time and waited outside of the testing area. This was done with the aim of keeping the exploring behavior as natural as possible. Participants were not aware of the purpose of the present study. During the interaction with the testing area, eye movement patterns as well as physiological activities were recorded with the mobile eye-trackers and E4 wristbands. Although there were no restrictions on how they walked the street, participants were not allowed to cross the street or enter shops. To evaluate the physiological activities precisely, participants were also asked not to run, eat, drink, or smoke during the walk. Participants were also asked not to use their personal phones. At the end of the 5 min, the researcher came back to the participant to stop the physiological and eye-tracking recordings.
3. *After the interaction with the testing area:* Right after the walk, the participants sat down and completed the subjective evaluations about the general experience, urban environment (PRS), and Graetzloase. Then, the physiological activities were measured for 5 min [data presented in Mikuni et al. (2024)]. After the physiological measurements, the participants came back to the experimental base and removed the equipped devices. Then, participants filled out another series of questionnaires evaluating their subjective wellbeing [data presented in Mikuni et al. (2024)] and individual differences (stress they felt over the past month, their nature-orientedness, and their art knowledge; these data were not analyzed in the present study). If it was their second and last visit, participants were debriefed and paid.

### 2.6 Pre-processing and data cleaning

**Figure 4:**
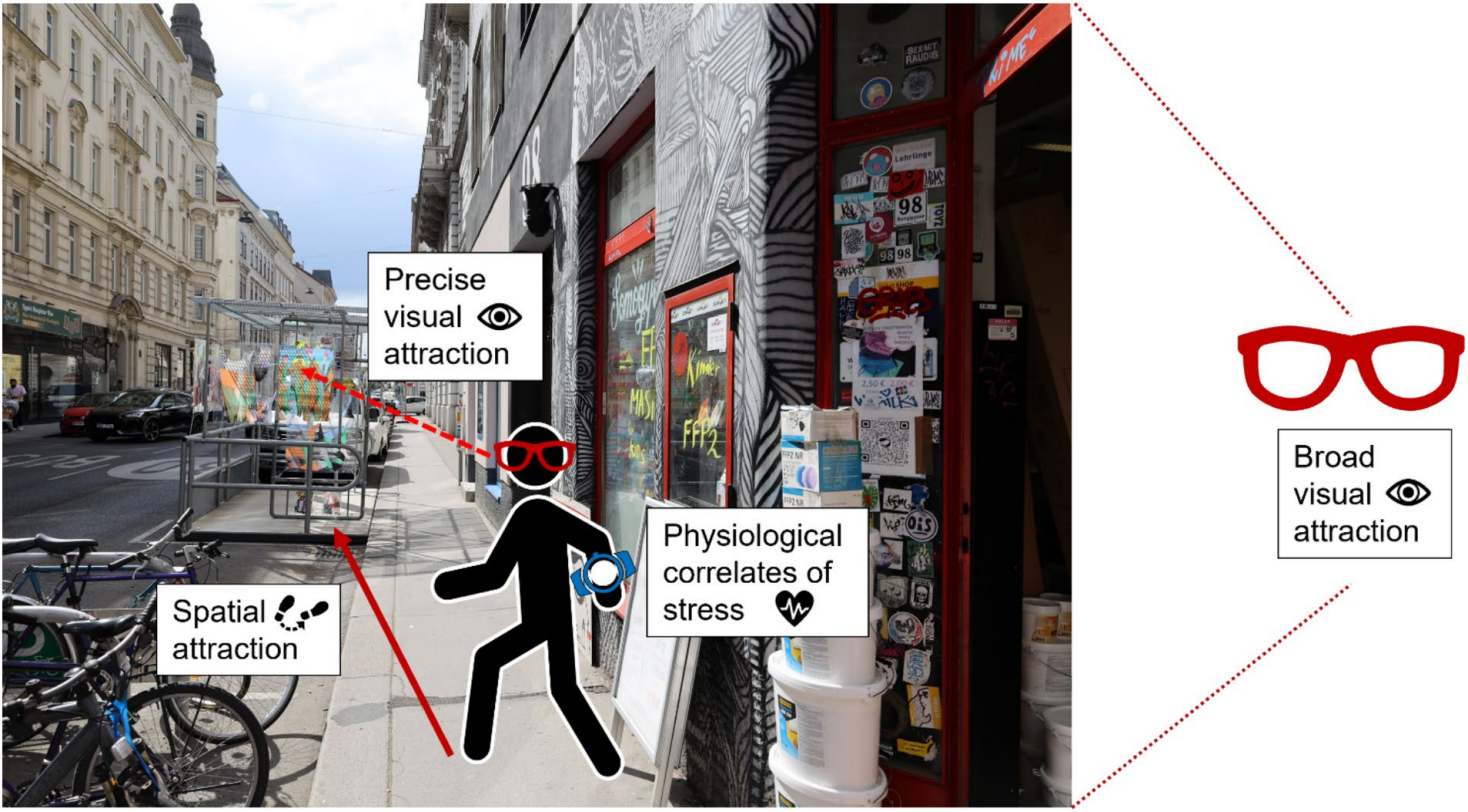
Visual representation of the four main dependent variables.

Before providing additional information about the individual variables, we wanted to highlight that during the data analysis process, it was noticed that a significant amount of gaze data did not have a good enough quality to be analyzed (see section “2.6.3. Final datasets” below for more information). Hence it was decided to include another measure of visual attraction, which did not rely on the precise gaze data and hence did not suffer from data loss. We refer to the measure of visual attraction based on the gaze data as *precise visual attraction* and the measure of visual attraction based on the eye-tracking video (and not on the gaze data) as *broader visual attraction*. This broader measure of visual attraction can be linked back to head movements, and together with the precise visual attraction, these measurements offer panoramic picture of the urban environment (Hillnhütter, 2021). A visual representation of the main dependent variables (DVs) that were used for the analyses (Broader visual attraction, Precise visual attraction, Spatial attraction—for attraction—and MOS scores for wellbeing) is provided in Figure 4.

#### 2.6.1 Eye-tracking pre-processing

The eye-tracking data were first processed with iMotions version 9.3 (iMotions, Copenhagen, Denmark). iMotions was chosen to perform the pre-processing of the data as it allows the combination of both Tobii Pro3 and Pupil Labs invisible data within the same environment. Detail information for the pre-processing is available in the supplementary material (see “2. Pre-processing and data cleaning: Detail information for the eye-tracking data pre-processing”). Three variables about the Graetzloase were extracted with iMotions. They are listed below:

1. Broader visual attraction: the time (in ms) the Graetzloase was present in the videos taken by the eye tracker field of view (FOV)^4^ camera. This time was obtained by manually annotating the eye-tracking videos when the Graetzloase was present in the FOV.
2. Precise visual attraction: To assess if and how long people directly look at the Graetzloase, we set the Graetzloase on the recorded videos as the area of interest (AOI). Total dwell time (ms) during which the participants gazed (fixation and saccade) on the Graetzloase was defined as precise visual attraction.
3. Spatial attraction: the time (in ms) participants spend inside the Graetzloase. This time was obtained by manually annotating the eye-tracking videos when the participants performed the behavior of being in the Graetzloase.

Note that we also extracted the times (in ms) that (1) green and (2) artistic elements external to the Graetzloase, (3) shop windows, and (4) passers-by on the street were present in the participant’s FOV. The times for these four specific elements of the streets were also obtained by manually annotating the eye-tracking videos. As these times are video-dependent and not gaze-dependent (contrary to the precise visual attraction of the Graetzloase), they also measure participants’ broad visual behavior.

The iMotions software produced different .csv file for the manual annotations and AOI metrics (i.e., we were interested in the total dwell time in the present case). All files were loaded into the R studio environment (version 4.3.1; R Core Team, 2023) to merge the data for the two visits of each participant. If the data for one visit were not found for one participant, an investigation was run. Upon this investigation, some participants were renamed, and others were excluded (see R analysis on the OSF page for more information about the investigation).

In the third step of the procedure (see “2.5 Procedure” above), we described the interaction that the participant had with the urban environment for 5 min. In reality, the walk did not last precisely 5 min. This is because, at the end of the 5 min, the researcher could have been far away from the participant’s position in the testing area. The time beyond the initial 5 min represents the duration it took for the researcher to reach the participant to end the step of interaction with the environment. At the stage of data analysis, we decided to end the walk at the moment when the researcher reached the participant to stop the wellbeing device. This was done in order not to cut the naturally occurring behaviors that happened during the time beyond the initial 5 min. This choice thus induced a “non-equal time of walk” for all the participants. To account for that, it was decided—for each participant—to divide all the timings in milliseconds (coming from the annotations and the AOI metrics) by the length of each individual walk. This created a ratio for each variable. The ratio values are comprised between 0 and 1, with numbers close to 0 representing low visual and spatial attraction and numbers close to 1 representing high visual and spatial attraction. We note that the three ratios created for (1) broader visual attraction, (2) precise visual attraction, and (3) spatial attraction were used as the main dependent variables in the following analyses using the eye-tracking data.

#### 2.6.2 Wellbeing pre-processing: the moments of stress (MOS)

The detection of the Moments of Stress (MOS) followed the procedure proposed by Moser et al. (2023), which is an advancement of the procedure established by Kyriakou et al. (2019). While Kyriakou et al. (2019) proposed a calculation of MOS based on general properties of the ST and EDA signal, Moser et al. (2023) found that ST is not suitable for acute stress detection in a time window of less than 10 s. Their method identifies MOS more reliably by tailoring the individual rules, which need to be met to detect a MOS, based on an individual’s baseline level of EDA. The MOS’s calculation was done as follows. Depending on the number of stress indicators, formulated as rules of the stress detection algorithm, which are met, a MOS score is added to the overall stress score. The maximum value for this stress score is 2.0. For our case, we chose an overall threshold of 1.25 to create a binary indicator variable, which differentiates between stress and non-stress. The higher we set this threshold, the more certain we can be about a detected stress situation, as there are more signs in the physiological reaction. Participants without one of their two visits recorded were identified, and the reason why they did not have two recordings was investigated. Depending on the case, participants were renamed or excluded (see R analysis on the OSF page for more information about the investigation). Participants that had their recording length strictly lower than 300 s (5 min) were removed.

Similarly, to the eye-tracking data, the length of the physiological recording for each participant was not the same. To account for the difference, a ratio between the number of seconds that were marked as a MOS (i.e., those with the binary indicator 1, indicating a detected stress situation based on Moser et al. (2023)), and the time of the walk was computed for each participant. This ratio of MOS was used as a main dependent variable for the analyses and labeled “MOS detected.” The ratio value is comprised between 0 and 1, with 0 meaning that the participant had no MOS during the walk (no stress) and 1 meaning that the participant was stressed during the entire walk.

#### 2.6.3 Final datasets

Participants who did not have the full dataset for both visits and for all types of data (i.e., the eye-tracking, wellbeing and subjective evaluations) combined were excluded. Moreover, due to variations in the quality of recorded gaze data among participants, two distinct datasets were generated for the analysis. More details about these datasets are provided below.

Although the calibration was done before recording the data, it was still possible that the data were not recorded properly due to the lighting conditions, movements, etc. This led to differences in the number of available data for the precise visual attraction and: (a) the broader visual attraction on the Graetzloase, (b) the broader assessment of the green and artistic elements external to the Graetzloase, shop windows, and passer-by on the street; (c) the spatial attraction. As these three listed elements (a to c) are based on the extraction of data from the eye-tracking videos, these data could be retrieved despite the quality of the gaze data. In other words, although the fixation/saccades were not recorded with high accuracy, information about these three listed elements (a to c) from the world view camera could be retrieved. For the three listed elements (a to c) as well as for the ratio of number of the MOS and the subjective evaluations, the data from 71 participants (Art-Street: *n* = 35, *M*_age_ = 24.38, *SD*_age_ = 5.70, Green-Street: *n* = 36, *M*_age_ = 24.35, *SD*_age_ = 7.33) were included in the analysis below.

On the other hand, the quality of the gaze data was critical to represent precise visual attention, as this was defined as the time when the participant’s gaze dwelled on the Graetzloase, hence requiring precise fixation/saccade information. Consequently, for the analyses involving the precise visual attraction, the data from 26 participants (12 in Art-Street and 14 in Green-Street) were included. Detailed data inclusion and exclusion criteria are provided in the R code uploaded on OSF.

## 3 Results

### 3.1 Descriptive statistics of the variables

A general overview of the descriptive statistics of the eye-tracking, wellbeing, and subjective evaluation variables is provided in Supplementary Table S3 of the supplementary material.

We note that participants had Moments of Stress (MOS) for about 3% of the time they interacted with the urban environment. This indicates that the participants have not been too stressed during the interaction with the testing area. Further interpretations will be provided in the discussion together with the inferential statistics.

Moreover, of the entire interaction time with the testing environment, participants on average spent between 1 and 3% of the time precisely looking at the Graetzloase. Regarding the broader visual attraction, the Graetzloase was present in the field of view between 35 and 46% of the time, on average. Regarding the spatial attraction: participants spent on average between 0 and 9% of the time inside the Graetzloase. This shows that the Graetzloase was relatively not very visually attractive, that it was relatively present in the participant’s visual field, and that people did not interact a lot with it. As for the MOS, further interpretations will be provided in the discussion together with the inferential statistics.

### 3.2 Planned analyses

A similar analytical structure was applied to see which effect art and greenery (present in the Graetzloase and on the street) would have on wellbeing (as measured by MOS) and on attraction (Broader Visual, Precise Visual, and Spatial). With main DVs, we ran a series of (generalized) liner mixed models [(G)LMMs]. The structure of the models was similar for the four DVs. The fixed effects were *Street* (Art-Street vs. Green-Street) and *Condition* (Green vs. Art). The interaction between the two factors was also added as a fixed effect. To consider the inter-individual differences (e.g., some participants might look at an object longer than the others regardless of the types of the objects, etc.), random intercept for participants was included as a random effect. A type 3 contrast was applied. As the data pre-processing step led to the creation of two datasets (*n* = 71 and *n* = 26), *a posteriori* power analysis was run with the “simr” R package (Green and MacLeod, 2016). Table 2 reports the *a posteriori* power for all the effects of the different models tested. For all the fixed effects of the models, the power varied between 5.70 and 83.00%. The power will be considered for the interpretation of the significant effects.

**Table 2:** *A posteriori* power calculation for each of the models.

	Effect size of the fixed effect	*A posteriori* power	95% confidence interval	Method
MOS model				
Street	−0.00	9.20%	[7.48, 11.16]	*z*-test
Condition	−0.00	6.10%	[4.70, 7.77]	*z*-test
Street × Condition	0.00	8.20%	[6.57, 10.08]	*z*-test
Broader visual attraction model				
Street	−0.04	28.30%	[25.53, 31.20]	*z*-test
Condition	0.00	5.70%	[4.35, 7.32]	*z*-test
Street × Condition	−0.07	59.60%	[56.48, 62.66]	*z*-test
Precise visual attraction model				
Street	0.73	39.60%	[36.55, 42.71]	*z*-test
Condition	−0.53	63.50%	[60.43, 66.49]	*z*-test
Street × Condition	−1.10	83.00%	[80.53, 85.28]	*z*-test
Spatial attraction model				
Condition	−0.31	54.90%	[51.76, 58.02]	*z*-test

Due to problems of R computation, the “contrasts = type3” was removed from the model formulas.

All analyses were performed using the “lme4” R package (Bates et al., 2015). The model output was assessed using the “summary” function from the R base and using the “effectsize” function from the “effectsize” R package (Ben-Shachar et al., 2020). We note that, depending on the assumption check as well as the type of the data, we adapted different distributions for our four dependent variables. This will be mentioned in the following sub-section and in the R scripts. All data as well as the R scripts are available on the OSF page. Finally, in the main text, we only reported detailed statistical information about significant results. Nevertheless, a full summary of the results is available in Supplementary materials (see Supplementary Tables S4–S7).

### 3.3 Assumption checks

For the MOS detected, data violated the normality assumption as tested by Shapiro–Wilk. But as the Q–Q plot of the residuals did not point towards a violation of normality, the sample size was superior to 30/40 (Ghasemi and Zahediasl, 2012), the participants were almost equally split (35 in Art-Street and 36 in Green-Street) in the two streets (between-subjects factor), and the homogeneity of variance was not violated for either factor, it was decided to carry on the analysis without applying any transformations.

For the broader visual attraction on the Graetzloase, the data violated the normality assumption as tested by Shapiro–Wilk. For the same reasons as the MOS detected, we did not transform the data.

For the precise visual attraction on the Graetzloase, the data also violated the normality assumptions (as evaluated by both Shapiro–Wilk and visual inspection). As the sample size of the precise visual attraction was below 30 (*n* = 26), the participants were less homogeneously split across groups (12/14 for the dataset of 26 participants compared to 35/36 for the dataset of 71 participants), and the homogeneity of variance assumption was not optimal for the between factor (*p*-Green-Street = 0.06), it was decided to use a GLMM to predict participant’s precise visual attraction.

For the spatial attraction, data violated normality (as evaluated by Shapiro–Wilk test) and homogeneity of variances for the between factor. The Q–Q plot of the residuals showed a severe deviation from normality. When looking at the repartition of the data points, it was noticed that in 94% of the Green-Street visits, the participants did not enter the Graetzloases at all. This fact created zero inflation, as when participants did not enter, they spent 0 min inside. On the other hand, participants entered the Graetzloases in only 51% of the Art-Street visits. This primary analysis brings information about the context (i.e., the street where the Graetzloases were placed). To investigate the impact of the Graetzloases itself, it was decided to only look at the data of the participants who entered the Graetzloases. In other words, we removed the participants who spent zero time inside the Graetzloases. This left the sample for Art-Street to 18 participants (36 visits) and the sample for Green-Street to 2 participants (4 visits). Due to the small sample size for Green-Street, it was decided to only analyze the data collected in the Art-Street. The assumptions were re-run. As they revealed a violation of normality, a GLMM was fitted to predict participant’s spatial attraction.

### 3.4 Aim 1: art, Graetzloase, urban environment, and wellbeing

**Figure 5:**
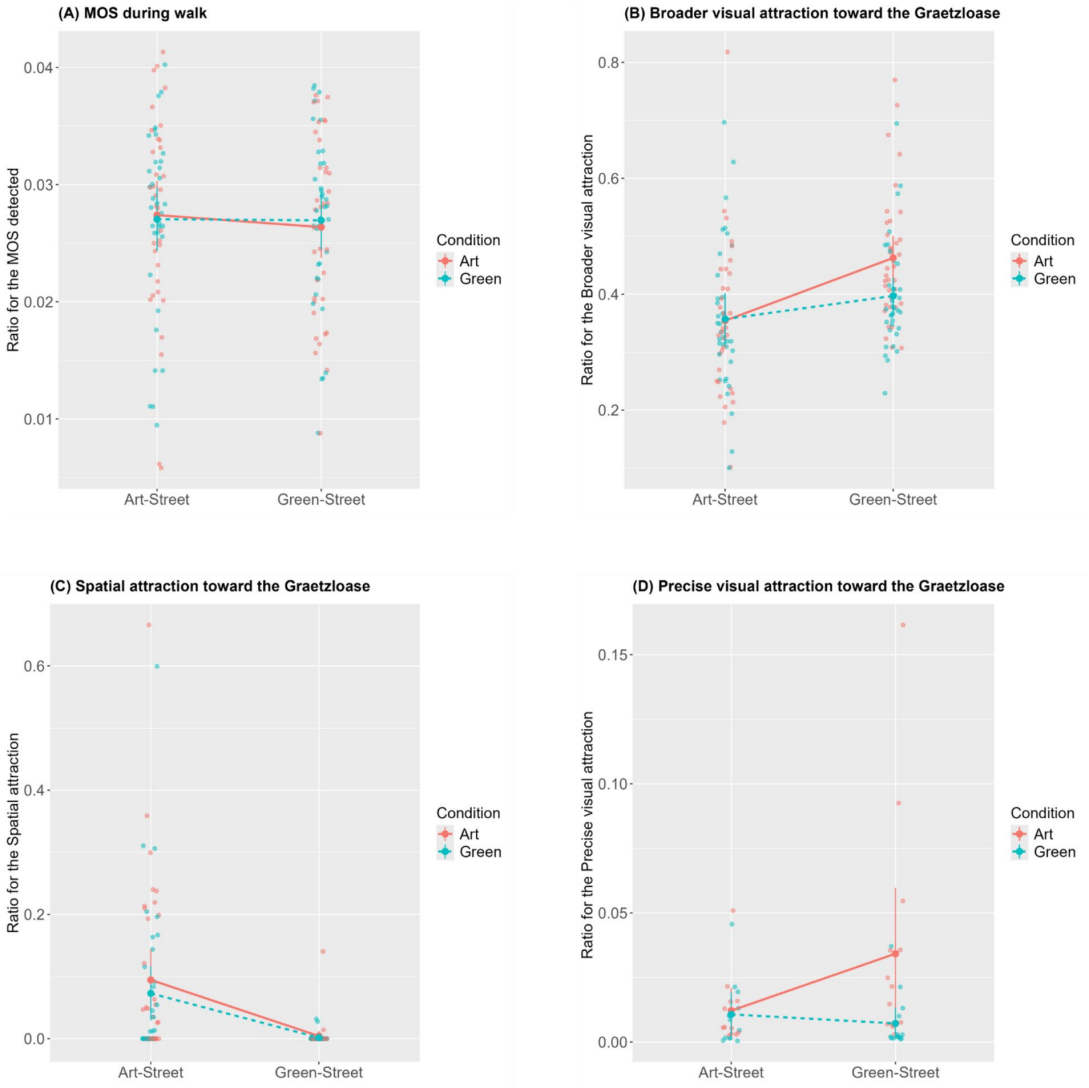
Means and 95% confidence intervals for the four main variables of interest. *Note.* These graphs represent the means of non-transformed data and not the estimated marginal means. The ratios expressed on the *y*-axes are the expression of **(A)** the number of moment of stress (MOS) the participants experienced, **(B)** the time the Graetzloase was present in the participants’ field of view, **(C)** the time the participants spent inside the Graetzloase, **(D)** the time the participants looked at the Graetzloase; over the time the participant took to interact with the urban environment (see 2.6 for more details).

An LMM was fitted to predict participant’s MOS detected by either the Street or the Condition. The analysis revealed no significant main effects or interaction (see Supplementary Table S4). A graphical visualization is reported in Figure 5A.

### 3.5 Aim 2: art, Graetzloase, urban environment, and attraction

#### 3.5.1 Broader visual attraction

A LMM was fitted to predict participant’s broader visual attraction. Our model showed a significant main effect of *Street* [*B* = −0.04, *p* = 0.002, *SE* = 0.01, *β* = −0.30, 95% CI (−0.48, −0.12)], a significant effect of *Condition* [*B* = 0.02, *p* = 0.043, *SE* = 0.01, *β* = 0.13, 95% CI (0.00, 0.25)], and a significant interaction between the two factors [*B* = −0.02, *p* = 0.028, *SE* = 0.01, *β* = 0.14, 95% CI (−0.26, −0.02)] (see Supplementary Table S5 for a full report of the statistics). Further interpretations of the results are provided in the supplementary material (see the section “5. Results: Aim 2: Art, Graetzloase, Urban environment and Attraction”). Based on the supplementary material and the graphical examination of the results presented in Figure 5B, there is reasonable evidence showing that the Art Graetzloase in the Green-Street was present in the participant visual field for longer times compared to the three other experimental scenarios [(1) Street: Art-Street, Condition: Art; (2) Street: Art-Street, Condition: Green; (3) Street: Green-Street, Condition: Green].

#### 3.5.2 Precise visual attraction

The choice of general distribution was assisted by the “fitdistrplus” R package (Delignette-Muller and Dutang, 2015). Two distributions were considered: Beta and Gamma. Even though the Beta distribution respected the ratio nature of our data (it is a distribution suitable for data expressed as proportion), we chose to implement the Gamma distribution with a log link function as it had a better fit. The fit was evaluated by comparing the AIC and BIC, and the Gamma had the smaller ones.

The GLMM was fitted to predict participant’s precise visual attraction. A significant main effect of *Condition* [*B* = 0.54, *p* < 0.001, *SE* = 0.10, *β* = 0.54, 95% CI (0.35, 0.74)] and a significant *Street* × *Condition* interaction [*B* = −0.27, *p* = 0.005, *SE* = 0.10, *β* = −0.27, 95% CI (−0.47, −0.08)] were found. No main effect of *Street* was found (see Supplementary Table S6). Further interpretations of the results are provided in the supplementary material (see section “5. Results: Aim 2: Art, Graetzloase, Urban environment and Attraction”). Based on the supplementary material and on the graphical examination of the results presented in Figure 5D, there is reasonable evidence showing that the Green Graetzloase in the Green-Street was looked at less than the Art Graetzloase in the Art-Street. There is also reasonable evidence showing that the Green Graetzloase in the Art-Street was looked at less than the Art Graetzloase in the Green-Street. Our results also show that participants looked for longer times at the Art Graetzloases than the Green one (regardless of the street they were put in).

#### 3.5.3 Spatial attraction

As for the precise visual attraction, the choice of general distribution was assisted by the “fitdistrplus” R package (Delignette-Muller and Dutang, 2015). Several distributions were considered (beta, gamma, lognormal, and Weibull), but the Gamma distribution was chosen as it had the best fit (evaluated via the AIC and BIC). This GLMM model was similar to the other ones (see the introductory paragraph of “3. 2 Planned analyses”), except that we removed the fixed effects of *Street* (Art-Street vs. Green-Street). The analysis revealed no main effect of *Condition* (Art vs. Green) (see Supplementary Table S7). A graphical visualization is reported in Figure 5C.

### 3.6 Aim 3: relationships between attraction, wellbeing, and subjective experiences

Regarding the exploratory analysis, spearman’s correlation scores were calculated between the broader and precise visual attraction, the spatial attraction, the MOS score, the general experience question (i.e., enjoyment, meaningfulness, duration), the PRS score, and aesthetic evaluations towards the Graetzloase (i.e., beauty, liking, meaningfulness, reflection). The plot was obtained with the “corrplot” function (Wei and Simko, 2021). To control for multiple comparisons, a Bonferroni correction of *p* = 0.00076 was adapted to evaluate the significance (*p* = 0.05 divided by 66 correlation tests).

**Figure 6:**
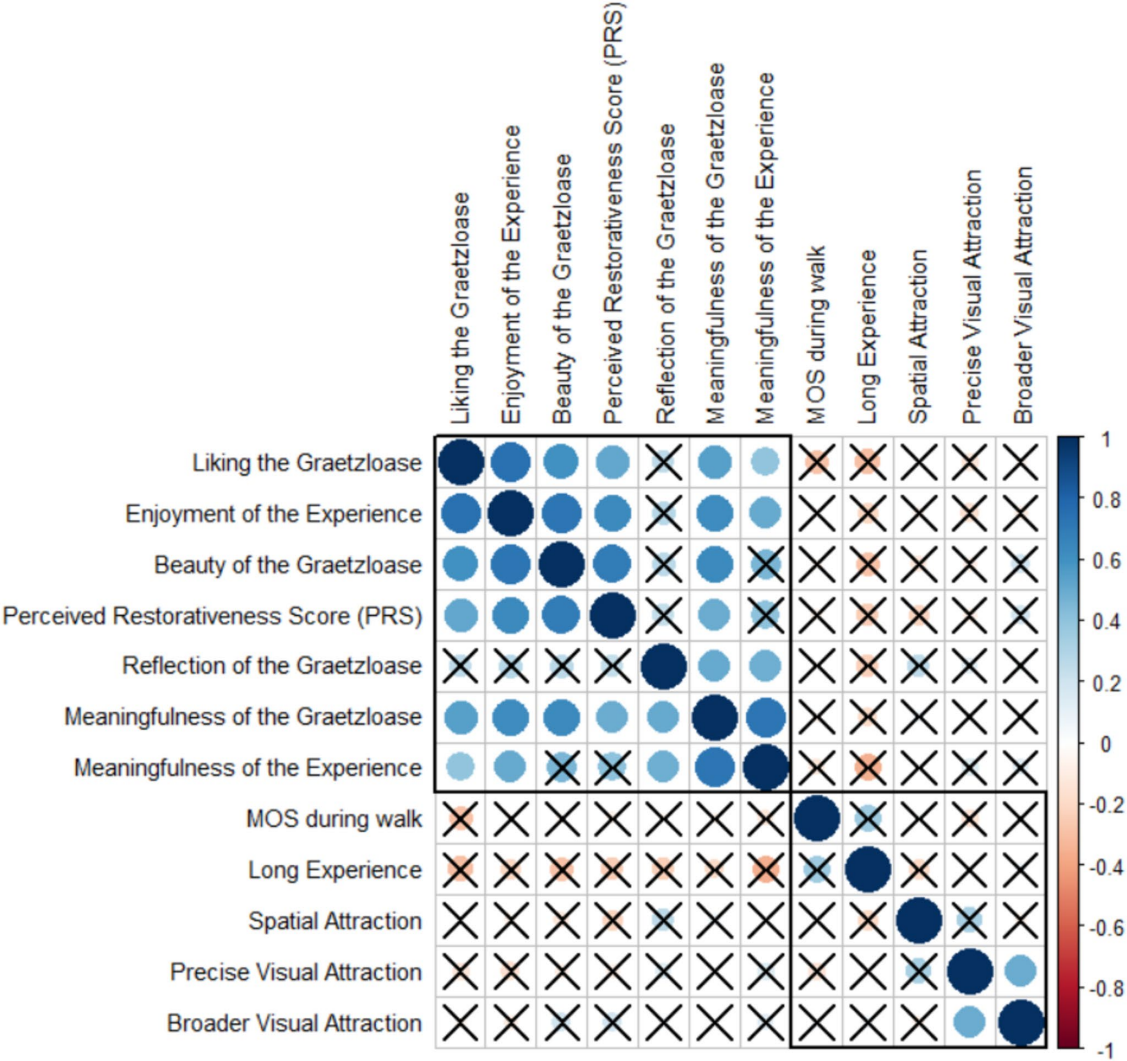
Correlations between attraction, wellbeing, and subjective evaluations. For this analysis, *N* = 26. This matrix collapses both Conditions (Green and Art) and both Streets (Art-Street and Green-Street). The blue color indicates a positive correlation, and the red color indicates a negative correlation. The size of the circles depicts the absolute value of the correlation coefficients. The crosses are drawn on the non-significant correlation (significance threshold: 0.00076).

Figure 6 shows the correlation plot. When looking at the expected links between attraction and aesthetic evaluations of either the experience or the Graetzloase, we report that none of the three attraction variables (broader visual attraction on the Graetzloase, precise visual attraction on the Graetzloase, spatial attraction towards the Graetzloase) were significantly correlated to the subjective aesthetic evaluations (Beauty of the Graetzloase, Liking the Graetzloase, Meaningfulness of the Graetzloase, Reflection of the Graetzloase, Enjoyment of the Experience, Meaningfulness of the Experience). Note that one significant positive correlation was found between the two variables representing the broader and precise visual attraction. There was no significant relationship between the variables representing aesthetics and wellbeing (MOS detected). Nevertheless, significant correlations between aesthetic evaluations of the experience or the Graetzloase and the restorativeness of the urban environment (PRS), which can be seen as an indicator of the wellbeing potential of an environment, were found. Finally, we note that most of the subjective aesthetic evaluation variables were significantly positively correlated together.

## 4 Discussion

In the present study, we explored how individuals perceive and experience their urban environment in which two parklet interventions (Graetzloases) were placed: one Graetzloase decorated with art was compared to one decorated with greenery (i.e., vegetation). In this field study, we captured participant’s real-time experiences on two distinctively different streets in Vienna. Our two main aims were to see: (1) if Art in the urban environment (i.e., framed in a Parklet and in a street) can impact wellbeing (2) if people would be attracted to this new type of urban element on the streets, especially if equipped with Art. As a pioneering study in the exploration of urban art and wellbeing, (3) we also investigated the different connections between attraction, wellbeing, and subjective experiences (participants’ general experience, the urban environment, and the Graetzloase).

Before diving into the discussion of the results related to each individual aim, we first wanted to comment on our choice of greenery as active control. Even if we provided evidence that the Green condition was a good active control for the Art (see the “2.2 Testing areas: streets and Graetzloases” part), we acknowledge that the question of finding a good control condition is a valid problem in the field studying the effects of wellbeing interventions in general (see Trupp et al. (2024) for a discussion on the matter). Future studies should replicate the present study also with the addition of a more conventional control condition (Graetzloases without art or greenery). In the future, one could also implement (clearer/more direct) check-up questions to see how the participant perceived the active control condition (at the end of the experiment). In our case, we could have asked, “Did you perceive the greenery as decoration?”

### 4.1 Aim 1: art, Graetzloase, urban environment, and wellbeing

Based on past studies, there is a rational to use art and greenery (either present in the Graetzloase or on the street) in a study measuring wellbeing as an outcome. This study presents the first results where stress (MOS) reactions as a response to art and greenery—used as a proxy for a person’s state of wellbeing—was tested. Hence, we primarily aimed to investigate whether artistic or green stimuli have the potential to elicit a (non)-negative stress response in an individual. The only working hypothesis that we had was: both Green and Art Graetzloases placed in the narrow and deprived of natural elements street would evoke more MOS compared to the two Graetzloases placed in the greener and wider street. Present descriptive results show that the MOS ratios were quite low in general (approximately 3%), indicating that the participants were not that stressed during their encounter with the urban environment. Inferential statistics did not show any significant differences between the *Condition* and the *Street*, the latter theoretically refuting our hypothesis. Nevertheless, cautiousness is required for the interpretation of these results, as all the fixed effects were largely underpowered (see Table 2). In other words, present results do not mean that there is no effect of the context as hypothesized, but simply that the present study does not have enough evidence to conclude. Future studies should replicate the present study with more participants to increase the reliability of the findings.

Nevertheless, one can still elaborate on other reasons why the expected effects were not there. For example, present results may also be explained by a floor effect. A floor effect happens when a large portion of the participants of an experiment score low on the variable measured, making it difficult to see differences between groups as most of the data points are already clustered at the lower end of the scale. The assumption was that test subjects came with a certain amount of stress (see Haider et al., 2023 for an example with a sample of Austrian students and Teixeira et al., 2020 for an example with an urban field experiment measure of MOS in cyclists), but the scores measured before the interaction with the testing area associated with the present dataset analyzed in Mikuni et al. (2024) show that subjects were more relaxed than we thought. Indeed, they experienced on average between 5.56 (expressed in ratio: 1.85%) and 6.68 (expressed in ratio: 2.23%), MOS over the 5 min their physiological activity was monitored before their encounter with the urban environment. The floor effect is as follows: If the participant, instead of being mildly stressed at the beginning of the experiment, was extremely relaxed, then it might be difficult to see the effect of elements (i.e., art and greenery in the Graetzloase) and/or structures (Graetzloase and the street it is embedded in) that were supposed to have a relaxing effect. Future research should consider the use of a stress-inducing protocol before the interaction with experimental conditions (see Keil et al., 2023 for an example with urban virtual reality and EDA).

Another possible implication of our findings is that the average 5-min interaction period that the participant had with the environment, might not have been long enough for a wellbeing effect to emerge, as this effect might require more time to manifest in such environments. What this reflection is touching upon is the notion of dose–response. A dose–response approach is interested in knowing what is the *Frequency*, *Duration*, and *Intensity* that is needed for an agent (that is thought to influence health) to have an effect, i.e., a larger stimulus might lead to effects with shorter exposure than a small stimulus (Cox et al., 2017). Cox et al. (2017) developed this approach with greenery and with subjective measures of wellbeing. Nevertheless, the combination of urban art and greenery stimuli and the MOS scores as evaluation of wellbeing is a novel approach. With a comparable experimental setup, future research could investigate the duration of contact with art and greenery; as well as the intensity of art and greenery that is needed to see an effect on wellbeing. The necessity of the second point (intensity) is further highlighted by our results: When looking at physiological reactions, the art and greenery might be too subtle compared to the sound of an air horn (the type of stimuli that the MOS algorithm was developed with). To also verify if the urban stimuli were too subtle to act on wellbeing, self-reported feedback from participants can be incorporated into future studies to cross-reference the psychophysiological response measured through wearable sensors and a person’s subjective description of their experience.

On the same note, if one wants to further study the interaction between the Graetzloases’ design and the context they are embedded in, one should consider placing the Graetzloase in more extreme streets. By extreme, we mean aesthetically deprived and aesthetically enhanced. The study of the potential impact of the Graetzloase (a structure that has both aesthetics and wellbeing potentials) on aesthetically deprived urban areas is interesting, as elements such as boarded-up buildings, graffiti, and litter were found to mediate poor wellbeing conditions for its inhabitants (Cohen et al., 2003). More precisely, studying the impact of an Art Graetzloase in deprived urban communities is even more interesting, as evidence showing that greenery positively impacts stress levels in such communities already exists (Roe et al., 2013; Ward Thompson et al., 2016).

### 4.2 Aim 2: art, Graetzloase, urban environment, and attraction

The first part of the second aim was about to see if people would be attracted to this new type of urban element on the streets. We postulated that the Graetzloases would be attractive elements of the street as they are salient and relatively novel. Our descriptive results show that out of the couple of minutes that participants spent in the vicinity of the Graetzloases, on average they only spent very little time (a couple of seconds) looking at it or spending time inside of them. Our hypothesis is refuted as present results indicate that the Graetzloases were not attractive. This could have been due to different reasons. First, it is possible that participants did not realize that the Graetzloase was a free, open-to-the-public space. This fact potentially accounts for our low occurrences of people entering the Graetzloases. Nevertheless, to avoid affecting the natural behavior and biasing the measurements, we refrained from explaining the function and purpose of the Graetzloase before the participant’s interaction with the urban environment. Given the fact that the Graetzloase is a relatively new concept, implementing an information sign indicating that Graetzloases are free for everyone could enhance city dwellers’ spatial attraction to them. Second, even though we tried to design attractive Graetzloases (e.g., the Art Graetzloases could have been visited like a small gallery, and the Green ones like a small greenhouse), these only contained either artworks or plants, and they were devoid of any amidites. In other words, they were “empty” in the sense that they did not offer any concrete utility for users (e.g., benches to sit on). Indeed, if a parklet seems empty, people might have realized quickly there is not much to do there and move on. Thus, the emptiness of the Graetzloases could (at least partly) explain why we obtain low levels of visual and spatial attraction. In reference to the study by Stevens et al. (2023), who worked on the playful usage of parklets, the impact of perceived utility of the Graetzloases on attraction—first, but also on wellbeing—could be an important element to further investigate. Future studies could implement benches and/or a more covering roof so that the Graetzloases could be seen as attractive structures that provide wellbeing by showcasing a possibility to sit, rest, protect oneself from the sun, and invite for other resting activities. Third, given that the urban environments, in which the two Graetzloases were embedded, were very stimulating (see Table 1 for the description of the streets and Supplementary Table S3 where the presence of other elements of the urban environment in the participant FOV are reported), it is possible that the visual attractiveness of the Graetzloases was overlooked by other attractive elements present in the street. For example, other eye-tracking studies show that participants’ attention is drawn more towards people, traffic, and the elements or aspects that are related to the act of walking (Chana et al., 2023; Foulsham et al., 2011; Mitschke et al., 2017). The fact that the two chosen Viennese streets for this study were already stimulating (e.g., they both had a lot of shops, and the Green-Street had prominent greenery, see Table 1) did not encourage the visual interaction with the Graetzloase. For future Graetzloases projects, we recommend an implementation of the structures in urban environments that are less stimulating.

The second part of the second aim was more precisely about the impact of the artistic urban intervention on attraction. Even though both Art and Green Graetzloase had a strong attraction potential, we hypothesized a strong effect of the context. Indeed, we thought that the contrast effect created by having an Art Graetzloase in a greener street and a Green Graetzloase in an artsy street, will visually and spatially attract the participants more towards the Graetzloases.

Before diving into the precise analysis of the three variables representing attraction (broader visual attraction, precise visual attraction, and spatial attraction), we would like to highlight again the following: Due to a loss of quality for some gaze data, we could not use all the collected data for the analysis of visual attraction, leading to a potential significant loss of power. It was thus decided to create a second variable to measure visual attraction that did not rely on the precise gaze data but on the video taken during the walk with the eye-tracking glasses. We called this new measure broader visual attraction, and called the one relying on the gaze data, precise visual attraction. Together, they offer a more complete representation of the participant’s visual attraction. During the data analysis step, the power was checked for each of the effects of broader and precise visual attraction as well as for the only effect of spatial attraction (see Table 2). All except one were underpowered (below 80%). Hence, these results require cautious interpretation, and further replication of the present study setup would be needed. Nevertheless, the effects that were decided to be focused on for the analysis (see the Supplementary Material for more information) had the most power. Indeed, the interaction for the broader visual attraction had 59.60% power; the interaction and the main effect of *Condition* for the precise visual attraction, respectively, had 83.00 and 63.50% power; and the main effect of *Condition* for the spatial attraction had 54.90% power. Moreover, this loss of data calls for the implementation of more check points during the collection of field data. Therefore, for future field data collections, we recommend researchers to implement an ongoing data quality check to ensure that the adequate number of datapoints for the data analysis steps are available (e.g., Bayesian approaches to optimising sample sizes for data analysis; Moerbeek, 2021).

#### 4.2.1 Broader and precise visual attraction

Even though all effects (main effects of *Street* and *Condition* and the *Street* × *Condition* interaction) were statistically significant for the broader visual attraction, we decided to focus on the interpretation of the significant interaction (see the Supplementary Material, section “5. Results” for Aim 2 for more information). Present results for the broader visual attraction partially support our hypothesis as we found a significant *Street* × *Condition* interaction; but we only found that the Art Graetzloase in the Green-Street was present for longer times in participants’ visual field. We did not find evidence for a longer presence in the visual field of the Green Graetzloase in the Art-Street. Results for the precise visual attraction support our hypothesis as we found a significant *Street* × *Condition* interaction. As for the broader visual attraction, the significant interaction that was obtained only partially supports our hypothesis: we found that Art Graetzloase in the Green-Street was more visually attractive (looked for longer times) than the Green Graetzloase in the Art-Street. We also found that there is reasonable evidence showing that the Art Graetzloase in the Art-Street was looked at for longer times than the Green Graetzloase in the Green-Street. This effect is linked to the main effect of *Condition* that we also found. A main effect of *Condition* means that a greater proportion of viewing behavior is dedicated to the Art Graetzloases than the Green Graetzloases regardless of the context. This can be explained by the fact that the contemporary art produced by our artists’ collaborators is a stimulating, unexpected, and compelling element when placed on the streets. Another explanation for this result can also be related to the amount of color in the different decoration sets. Indeed, while the Green decoration offered only one main color to see (green), the art set was more colorful, potentially attracting more visual attention. One can thus question the fact that the present effect is due to the fact that colorful decorations are labeled as art or because the art decoration were colorful in nature. In other words, would the results and conclusions regarding the art effect be the same if the art presented was in black and white? To further unpack the effect of art displayed in urban environments, future research should present a different variety of artworks containing different visual characteristics (e.g., of aesthetic visual properties that impact visual perception: color, shapes, symmetry, etc.).

When looking at the evidence brought from the analyses of both variables representing visual attraction together (broader and precise), we can say that they are going towards the same direction. Together, these results (even though underpowered) show that implementing art is visually attractive, but also that its attraction depends on the urban environments in which the art is implemented. When selecting the streets, it became apparent that the Green-Street streetscape might have been more visually boring compared to the Art-Street (see Table 1). Indeed, even though it had prominent greenery, the Green-Street had no decorative buildings, and all facades looked the same. Yet, in this setup, the Art Graetzloase was more visually attractive than the Green (for both broader and precise visual attractions). We attributed this to the contrast between the art in the Graetzloase and the greenery on the street. This concept is discussed by Hillnhütter (2021), when she concludes about the importance of making non-monotonous urban environments with a human scale to be appealing to the pedestrians’ senses. Nevertheless, in the Art-Street, where the building facades were way less visually boring, the contrast effect was not found. This strengthens our previous recommendation to implement Graetzloase in urban environments that are less stimulating.

#### 4.2.2 Spatial attraction

Due to the nature of the data, we were not able to assess the contrast effect for the spatial attraction with inferential statistical evidence. Nevertheless, our findings regarding people entering the Graetzloase in the first place, while not informing us on the contrast hypothesis, still allow us to draw conclusions on the impact of context. Participants in the Green-Street entered the Graetzloase in only 6% of the visits and entered the Graetzloase in 51% of the visits in the Art-Street. One can therefore infer that the street context impacts the Graetzloases’ spatial attraction, which could be explained by the spatial streets’ characteristics. As the Art-Street is a narrower street (see the “Enclosure and Accessibility (physical/visual)” line in Table 1), it is possible that participants were more prone to use the extra public space that the Graetzloase offered in order to avoid colliding with other people (Jovancevic et al., 2006). In contrast, the Green-Street is a wider street; therefore, participants would have ample space and not need to enter the Graetzloase. Our interpretation based on the street spatial characteristics is supported by the non-significant (yet, underpowered) main effect of *Condition*: no matter what is displayed in the Graetzloase in the Art-Street, people will spend time inside of it as they might lack space on the street.

#### 4.2.3 Visual and spatial attraction

Following on the last comment about the interpretation of the spatial attraction only, we wanted to highlight that the spatial arrangement of the street could have been also a reason why participants looked at the Art Graetzloase in the Green-Street. Indeed, as the Green-Street was way larger than the Art-Street, one could say that they had more space to visually engage. On the other hand, the narrower Art-Street offered less space to visually interact with the Graetzloase. This conclusion brings new evidence to the discussion on the design of (visually) stimulating urban environments at a human scale (i.e., a scale that deals with a couple of metres, proportionate to the size of humans) started by Hillnhütter (2021).

### 4.3 Aim 3: relationships between attraction, wellbeing, and subjective experiences

In a set of exploratory analyses, the relationships between visual/spatial attraction, wellbeing, and participant’s subjective evaluations were analyzed. Even though this analysis was exploratory, we had certain expectations based on previous literature. We expected that attraction (both visual and spatial) towards the Graetzloase would significantly and positively correlate with the subjective aesthetic evaluations of the Graetzloase. Present results do not support this expectation and disagree with past literature showing that longer viewing times are positively linked to aesthetic evaluations [see Mitschke et al. (2017) and de la Fuente Suárez (2020); for examples in an urban context]. Nevertheless, recent evidence also shows that, in the context of a free-viewing urban exploration, people’s aesthetic evaluation of street signs did not correlate with the viewing time (Chana et al., 2023). On top of all the explanation that are linked to attraction of the context the Graetzloase were placed in (see above parts of the discussion), we explain this result by the fact that Mitschke et al. (2017) and de la Fuente Suárez (2020) for their correlation analysis gave their participants more opportunities to look at urban elements they were interested in. In the case of the present study, only one Graetzloase was present in the street, and it was relatively small compared to the size of the area participants could freely explore. Bojko (2013) also mentions this concept, observing that certain elements in the environment can attract attention because of the “visual prominence of the area” standing out from the surrounding environment. Moreover, as the past literature linking attraction and aesthetic evaluation is focused on objects that can be seen and not objects that can be interacted with, our result of the spatial attraction towards the Graetzloase and its aesthetic evaluation brings new insight to the literature looking at aesthetic evaluations of urban environments. Based on previous literature, we also expected a positive correlation between the physiological correlates of wellbeing and: (1) the subjective aesthetic evaluations of the general experience; and (2) the subjective aesthetic evaluations of the Graetzloase. These correlations were not found, as the number of MOS was not significantly correlated to any of the six aesthetic evaluations. This result might be explained by the fact that past literature reporting a link between aesthetics and wellbeing is based on subjective evaluation for the wellbeing (Galindo and Hidalgo, 2005; Galindo and Rodriguez, 2000; Mikuni et al., 2024), which is not the case here as the MOS are a physiological correlate of wellbeing. It would therefore seem that wellbeing, when subjectively measured, is different from wellbeing as measured by an objective physiological metric and that these two measures behave differently when correlated to subjective measures of aesthetics (see Goyal et al.’s (2016) review for a list of advantages and disadvantages between objective and subjective measures of stress).

### 4.4 Conclusion

To summarize, our study revealed that the type of tested parklet had a relatively low attraction on people. Nevertheless, the Art Graetzloases were found to be more visually attractive (precise visual attraction) than the ones equipped with greenery. A contrast effect was found for the visual attraction (both broader and precise): our results revealed that the contrast of the Art Graetzloase against the wide street with urban greenery is more visually attractive than a Green Graetzloase in a narrow street with art murals. The street in which the Graetzloase is embedded also impacts participants’ spatial attraction towards them, with narrow streets prompting more participants to use them. Physiological indicators of wellbeing, as measured by an average of MOS, did not differ according to the street or type of Graetzloase.

To conclude, our results empirically inform on *What* to put in the Graetzloase as well as *Where* to implement the Graetzloases in the cities. This study, which shows that public art framed in an urban intervention attracts people’s visual attention alone, and that this effect is stronger in certain contexts, could be used as a starting point for a more evidence-based design of future parklets.

## Data Availability

The datasets produced and analyzed for this study as well as supporting documents can be found in the Open Scientific Framework (OSF): https://osf.io/qbcy7/.

## Footnotes

^1^Note that from here on when we refer to Art and Green with a capital A and G we are referring to the conditions of our experiment (the decorations in the Graetzloase). Green stands for greenery and Art for artworks.

^2^Note that the descriptive statistics provided here, for the age, are for *N* = 128, as two participants did not provide their age.

^3^Note that these data are provided for the 71 participants that were retained for the analysis (for more information, see "2.6.3 Final datasets").

^4^Note that we approximate the camera FOV as the participant visual field.
