# Supplementary material for: Exploring the influence of urban art interventions on attraction and wellbeing: an empirical field experiment
Source: Front Psychol. 2024 Dec 5;15:1409086. doi: 10.3389/fpsyg.2024.1409086 (PMC11656315; doi:10.3389/fpsyg.2024.1409086)
Supplement: Supplementary file 1 [file Data_Sheet_1.docx]

Supplementary Material

# Material: Main characteristics of the Eye Tracking devices

Table S1 presents the main characteristics of the two eye tracking devices used in the data collection. Two devices were used to increase the possibilities of testing more participants in a day. Indeed, our protocol allowed to have overlap between two participants sessions (i.e., when participant 1 was doing the post measurement phase (see Mikuni et al., 2024), still equipped with the first eye-tracker, participant 2 was equipped with the second eye-tracker at the experimental based in the meantime). To report the technical details, we followed the guidelines provided by Holmqvist et al. (2021).

| **Supplementary Table S1**  ***Technical details about the two eye tracking devices used*** | | |
| --- | --- | --- |
| Manufacturer | Tobii Technology | Pupil Labs |
| Model name | Tobii pro glasses 3 | Pupil Invisible |
| Eye-tracking Technique | Corneal reflection (dark pupil)  Stereo geometry | Corneal reflection (dark pupil)  Real-time neural network |
| Filters (built in) | Slippage Compensation: 3D eye tracking model. | Slippage Compensation: Slippage invariant. Deep Learning powered. |
| Software used to record the data | Real time and post hoc: Glasses 3 app | Real time: Pupil Invisible Companion app  Post-hoc: Pupil Cloud |
| Eye camera characteristics | 4 x eye cameras  16 illuminators  50Hz | 2 x Infra-Red eye cameras  192 x 192 at 200 Hz |
| Scene (Field of view) camera characteristics | 106° view in 16:9 format diagonal (H: 95°, V: 63°)  Format and resolution: 1920 x 1080 at 25 frame per second. | 82°x82° Field Of View  Format and resolution: 1088 x 1080px at 30Hz. |
| Sources of information | Information obtained from *Tobii Pro Glasses 3: Latest in wearable eye tracking^[[1]](#footnote-1)^* and *Tobii Pro Glasses 3 User Manual^[[2]](#footnote-2)^* | Information obtained from *Pupil invisible - eye tracking glasses technical specifications^[[3]](#footnote-3)^* and *Technical overview^[[4]](#footnote-4)^* |

# Pre-processing and data cleaning: Detail information for the Eye tracking data pre-processing

Two different strategies were adopted to analyse the eye tracking data: (1) a video-based analysis and (2) a gaze-based analysis. Both analyses were run because, due to the field nature of our study, it was difficult to acquire good gaze data quality. This made the gaze-based analysis difficult to perform. Hence, this is why we also did a broader analysis of the elements present in each participant’s video. We report the details for each analysis below as well as more information about the ratters (also referred as coders) who performed these analyses (following Holmqvist et al., 2021’s guidelines).

## Video-based analysis: Manual annotations

A video-based analysis was performed for the participants that had a correctly recorded video for both visits. This was done to respect the within factor *Condition* (Art Graetzloase vs. Green Graetzloase) of the inferential statistical analyses performed. In the video-based analysis, the camera Field Of View (FOV) was treated as an approximation of the human visual field. The presence of the Graetzloase, shop windows, people, greenery (external to the Graetzloase), and art (external to the Graetzloase) the in the participant’s visual field were annotated. An annotation is defined by the length of time (in millisecond) that the behaviours or elements were present in the participant visual field. Table S2 reports the descriptions of the annotated behaviours/elements. Coders were asked to code the videos from the moment the experimenter pressed start on the physiology phone until the experimenter came back to the participant after the five minutes of free exploration.

| **Supplementary Table S2**  ***Definition and characteristics of the different behaviours and elements that were annotated.*** | |
| --- | --- |
| **Behaviour / Element** | **Definition / Characteristics** |
| Graetzloase^a^ | When the Graetzloase occupied approximately 10% of the camera field of view. This 10% of the camera field of view situation could happen when:   - In Burggasse: The participant was between the garage entrance and the door with the glass (empty space between Halasz and Terra Vaper). - In Maria-Tusch-Strasse: The participant was between the end of the mobile shop and the bank. |
| Non-Graetzloase Art | Time when art (external to the Graetzloase) was on the camera field of view. This annotation did not have any percentage of screen occupation criteria (i.e., if art was on the video, coders made an annotation). We defined as art an element which was deliberately created as an art object.  List of the elements that were and were not considered as art in Burggasse:   - Were considered as art elements: The murals on the building façade, the art inside the BG98 shop, the graffiti between the BG98 and Terra Vaper shops. - Elements that were not considered as art: Art equipment in the Somgyi shop, the figurines in the Japanese martial art club house, the stickers on the traffic sign.   List of the elements that were and were not considered as art in Maria-Tusch-Strasse:   - Were considered as art elements: None. - Elements that were not considered as art: The ad for the insurance company. |
| Non-Graetzloase Green | Time when greenery (external to the Graetzloase) was on the camera field of view. This annotation did not have any percentage of screen occupation criteria (i.e., if greenery was on the video, coders made an annotation). We defined as greenery any natural elements such as plant, flowers, trees, bushes ect....  List of the elements that were considered as greenery in Burggasse: This street only had two elements that were counted as greenery: (1) The plants in the Terra Vaper shop window, (2) the terrace of the bar after Halasz hairdresser had some flowers on some videos.  List of the elements that were considered as greenery in Maria-Tusch-Strasse: All types of vegetation were counted as greenery. Note that greenery in the reflection of a window was also annotated as greenery. |
| Passer-by | Time passers-by (also referred as people or persons) are present on the camera field of view. The distance did not matter for counting the people (i.e., they could have been very close to the participant or faraway). All human persons on the street were considered as people. Note that, a person a with a stroller or a bicycle and the reflection of person in the shop windows were also counted as people. The experimenter was never counted as people. |
| Shop Windows | Time when shop windows occupy at least 10% of the camera field of view. Note that in some cases, the shop window contained greenery or artistic elements (e.g., Terra Vaper is a shop window with green, the BG98 is both shop window with art). In these cases, coders used two annotations for the same event (i.e., shop window and greenery; shop window and art). Shops which were on the other side of the street were not counted. Outdoor shop signs visible from far away were not counted as shop windows as they were not considered as a part of the shop façade line.  List of the elements that were and were not considered as shop windows in Burggasse:   - Were considered as shop window: Japanese martial art club house, Somgyi, Burggasse 98 (allsoldout shop), Tera Vaper, Halasz hairdresser. - Were not considered as shop window: Hel-Wacht green sign, Burggasse 96 area (the glass door, the garage and the white door), Burggasse 100 and 100a (Residential façade).   List of the elements that were and were not considered as shop windows in Maria-Tusch-Strasse:   - Were considered as shop window: Fix mobile (4 glasses in total), Winner Stadtische (4 glasses in total), Erste Bank window (6 glasses in total). - Were not considered as shop window: Building entrance (building Maria-Tusch-Strasse 19). |
| Entering the Graetzloase^b^ | When the participant stepped into the Graetzloase. |
| Comments (not analysed in this the present paper) | This annotation line was used when the coder wanted to make specific comments about an event. Here are some examples:   - If the participant touched the Graetzloase - Unusual events, such as: Talking to people, bike in the sidewalk (a bike on the road is traffic), dogs, babies… - Forbidden behaviours, such as: Entering the shops, crossing the street, entered the sandy parts (in Maria-Tusch-Strasse) |
| *Notes.* ^a^ The element “Graetzloase” will be the foundation of the variable called “Broader visual attraction” in the Table S3. ^b^ The behaviour “Entering the Graetzloase” will be the foundation of the variable called “Spatial attraction” in the Table S3. | |

## Gaze-based analysis: The Graetzloase as AOI

The gaze-based analysis consisted in drawing Area Of Interest (AOI) on a moving image. The obtention of AOI metrics (i.e., total dwell time in the present case) was based on the gaze data collected during the experiment and could only be performed for a subset of the participants who had good gaze data presence for both visits.

The percentage of data presence provided by iMotions was used a general indicator of the gaze data quality. Data recoded by the Pupil Invisible Glasses had 100% of data presence. This is due to the way the glasses record the data (see Tonsen et al., 2020). All Pupil Invisible videos were considered for the AOI analysis. On the other hand, Tobii Pro Glasses 3’s data presence values were scattered. Thus, we first looked at the videos with the highest percentage of data presence. Then, on a case-by-case basis videos were assessed based on a decreasing data presence percentage value. It was noted that below 70% of data presence, the gaze quality started to deteriorate.

Nevertheless, the decision to analyse or not the gaze data was not only based on percentage of data presence. Indeed, Moments Of Interest (MOI) were defined when the gaze of the participant was very close to the Graetzloase (the AOI) in the video recordings. If data points were missing during these moments, then the data was not analysed. To check for missing data point, we first looked on the video if the gaze trace disappeared. In addition, we also looked at Gaze 2D image (X and Y coordinates) as well as the fixation duration signals. When the Gaze 2D signals dropped, it meant that signal was not present anymore. This phenomenon was also reflected by a drop in the fixation duration histogram.

For selected videos, coders drawn a square on top of the Graetzloase. This square was the AOI. The drawing of the AOI was done in a liberal way (i.e., the AOI shape was not perfectly fitting the outline of the Graetzloase as that depending on the angle of view the Graetzloase was not a perfect rectangle). The square was drawn when the participant’s gaze was on the Graetzloase. Two specific behaviours were not considered as AOI: (1) when people were watching the Graetzloase through the reflection of the window and (2) when the participant looked behind the Graetzloase (e.g., when the participant looked at a passer-by in between two artworks). Note that AOI on a moving image analysis was only done for the Graetzloase and not for the other elements listed in Table S2 (e.g., art and greenery in the context) as the main interest of the paper is to see how people react to the interventions. Finally, note that, as for the video-based analysis (annotations), the within participant design was respected (i.e., only the participants with videos with good data quality for both visits were analysed).

Several parameters were fixed for the extraction of the final AOIs metrics. As the data sampling frequencies were between 50 and 200 Hz (relatively low), duration dispersion filter (I-DT) was applied (Holmqvist, 2011, p.153). No gap fill-in (interpolation) as well as noise reduction filter were applied. The threshold for dwell classification was set to 100 ms, which is what was typically reported in other eye tracking studies (Punde et al., 2017; Salvucci and Goldberg, 2000). First fixations as well as fixations starting before AOI onset or ending after AOI offset were not excluded.

## Inter-rater agreement

Three coders assisted with the pre-processing of the eye tracking data. They received the same instructions before the manual coding sessions began. The inter-rater agreement was verified at the end of the coding sessions via the calculation of a Cohen’s Kappa. It is a commonly used statistics to determine the inter-rater agreement on a nominal scale (Warrens, 2015). The procedure for the Cohen’s Kappa calculation is as followed: The three coders had to re-code the same minute from one video. Every 50 ms per 50 ms coders had to indicate, with 1 for *yes* and 0 for *no*, if they would annotate a specific behaviour/element or if they would activate the moving AOI. Note that Coders 1 and 2 both coded annotations and AOIs, whereas Coder 3 only coded AOIs. The Cohen’s Kappa calculations were performed with Microsoft excel (Microsoft Corporation, 2018). Cohen’s Kappa values between Coders 1 and 2 and for the annotations were: Graetzloase (.973), Non-Graetzloase Art (.825), Non-Graetzloase Green (.969), Passer-by (.864), Shop Windows (.927), and Entering the Graetzloase (1). For the AOI, Coders 1 and 2 had a Cohen’s Kappa of .819. Coder 3 had a Cohen’s Kappa of .732 with the Coder 1 and had a Cohen’s Kappa of .561 with the Coder 2. According to the guidelines reported in Landis & Koch (1977), Coders 1 and 2 had an almost perfect agreement as all their Cohen’s Kappa values were within a .81-1.00 range. Coder 3, who only coded AOIs, had a good substantial with coder 1 (substantial agreement range: .61-.80) and a moderate agreement with coder 2 (moderate agreement range: .41-.60).

# Descriptive statistics of the Eye tracking variables, Wellbeing variable, and Subjective evaluation variables

Table S3 reports the descriptive statistics of the eye tracking, wellbeing, and subjective evaluations variables. The eye tracking variables as well as physiological wellbeing measurement are represented by a ratio which lies between 0 and 1 (see section “2.6.1. Eye tracking pre-processing” and “2.6.2. Wellbeing pre-processing: the moments of stress (MOS)” in the manuscript for more information about the ratio calculation). The subjective evaluations are represented by seven-point Likert scales (see section “2.4.3. Subjective evaluations of the general experience, urban environment, and Graetzloase” of the manuscript for more information).

| **Supplementary Table S3**  ***Means, Standard Deviations (SD) and 95% Confidence Intervals (CI) for all the variables.*** | | | | | | | | | | | | | |
| --- | --- | --- | --- | --- | --- | --- | --- | --- | --- | --- | --- | --- | --- |
|  |  | Art-Street (Burggasse, BG) | | | | | | Green-Street (Maria-Tusch-Strasse, MT) | | | | | |
|  |  | Art Graetzloase | | | Green Graetzloase | | | Art Graetzloase | | | Green Graetzloase | | |
|  | | Mean | SD | 95% CI | Mean | SD | 95% CI | Mean | SD | 95% CI | Mean | SD | 95% CI |
| Eye tracking variables | | | | | | | | | | | | | |
|  | About the Graetzloase | | | | | | | | | | | | |
|  | Broader visual attraction | 0.35 | 0.13 | [0.31, 0.40] | 0.36 | 0.13 | [0.31, 0.40] | 0.46 | 0.11 | [0.42, 0.50] | 0.40 | 0.09 | [0.37, 0.43] |
|  | Precise visual attraction* | 0.01 | 0.01 | [0.00, 0.02] | 0.01 | 0.01 | [0.00, 0.02] | 0.03 | 0.04 | [0.01, 0.06] | 0.01 | 0.01 | [0.00, 0.01] |
|  | Spatial attraction | 0.09 | 0.14 | [0.04, 0.14] | 0.07 | 0.13 | [0.03, 0.12] | 0.00 | 0.02 | [-0.00, 0.01] | 0.00 | 0.01 | [-0.00, 0.00] |
|  | About the broad urban environment | | | | | | | | | | | | |
|  | Non-Graetzloase Art | 0.46 | 0.11 | [0.42, 0.50] | 0.48 | 0.09 | [0.44, 0.51] | 0 | 0 | [0, 0] | 0 | 0 | [0, 0] |
|  | Non-Graetzloase Green | 0.12 | 0.07 | [0.10, 0.14] | 0.12 | 0.05 | [0.10, 0.14] | 0.97 | 0.04 | [0.95, 0.98] | 0.98 | 0.03 | [0.97, 0.98] |
|  | Passer-by | 0.51 | 0.10 | [0.48, 0.55] | 0.50 | 0.15 | [0.45, 0.55] | 0.84 | 0.12 | [0.80, 0.88] | 0.88 | 0.08 | [0.85, 0.90] |
|  | Shop windows | 0.54 | 0.12 | [0.50, 0.59] | 0.53 | 0.12 | [0.49, 0.58] | 0.54 | 0.16 | [0.48, 0.59] | 0.53 | 0.17 | [0.47, 0.58] |
| Wellbeing variable | | | | | | | | | | | | | |
|  | MOS detected | 0.03 | 0.01 | [0.02, 0.03] | 0.03 | 0.01 | [0.02, 0.03] | 0.03 | 0.01 | [0.02, 0.03] | 0.03 | 0.01 | [0.02, 0.03] |
| Subjective evaluations variables | | | | | | | | | | | | | |
|  | About the Experience | | | | | | | | | | | | |
|  | Enjoyment | 4.97 | 1.46 | [4.47, 5.47] | 4.69 | 1.25 | [4.25, 5.12] | 5.06 | 1.17 | [4.66, 5.45] | 5.08 | 1.36 | [4.62, 5.54] |
|  | Meaningfulness | 4.43 | 1.60 | [3.88, 4.98] | 4.60 | 1.48 | [4.09, 5.11] | 4.47 | 1.40 | [4.00, 4.95] | 4.33 | 1.49 | [3.83, 4.84] |
|  | Long | 1.97 | 1.60 | [1.42, 2.52] | 1.51 | 0.92 | [1.20, 1.83] | 1.86 | 1.27 | [1.43, 2.29] | 2.31 | 1.56 | [1.78, 2.83] |
|  | About the Urban environment | | | | | | | | | | | | |
|  | PRS scores | 3.40 | 0.82 | [3.11, 3.68] | 3.34 | 0.96 | [3.01, 3.67] | 3.72 | 0.83 | [3.44, 4.00] | 3.71 | 0.87 | [3.41, 4.00] |
|  | About the Graetzloase | | | | | | | | | | | | |
|  | Beauty | 4.46 | 1.46 | [3.95, 4.96] | 4.26 | 1.34 | [3.80, 4.72] | 4.89 | 1.19 | [4.49, 5.29] | 4.89 | 1.19 | [4.49, 5.29] |
|  | Liking | 5.00 | 1.37 | [4.53, 5.47] | 4.83 | 1.20 | [4.42, 5.24] | 5.25 | 1.18 | [4.85, 5.65] | 4.97 | 1.11 | [4.60, 5.35] |
|  | Meaningfulness | 3.43 | 1.36 | [2.96, 3.89] | 3.83 | 1.60 | [3.28, 4.38] | 3.33 | 1.55 | [2.81, 3.86] | 3.50 | 1.30 | [3.06, 3.94] |
|  | Reflection | 4.09 | 1.50 | [3.57, 4.60] | 4.46 | 1.56 | [3.92, 4.99] | 4.22 | 1.79 | [3.62, 4.83] | 4.44 | 1.56 | [3.92, 4.97] |
| *Notes.* * For the precise visual attraction, the reported descriptives are from the smaller data set (*N* = 26). All the other descriptives for the different variables are based on the bigger data set (*N* = 71). | | | | | | | | | | | | | |

# Results: Aim 1: Art, Graetzloase, Urban environment and Wellbeing

**Supplementary Table S4**

***Full report of the Moment of Stress (MOS) model’s result.***

|  |  | B | *SE* | *t(1.380e+02)* | *p* | *β* | 95% CI |
| --- | --- | --- | --- | --- | --- | --- | --- |
| Fixed effects | |  |  |  |  |  |  |
|  | Street | 0.00 | 0.00 | 0.43 | 0.67 | 0.04 | [-0.13, 0.20] |
|  | Condition | -0.00 | 0.00 | -0.09 | 0.93 | -0.01 | [-0.17, 0.16] |
|  | Street*Condition | 0.00 | 0.00 | 0.35 | 0.73 | 0.03 | [-0.14, 0.20] |

# Results: Aim 2: Art, Graetzloase, Urban environment and Attraction

## Broader visual attraction

**Supplementary Table S5**

***Full report of the Broader Visual Attraction model’s result.***

|  |  | B | *SE* | *t(69)* | *p* | *β* | 95% CI |
| --- | --- | --- | --- | --- | --- | --- | --- |
| Fixed effects | |  |  |  |  |  |  |
|  | Street | -0.04 | 0.01 | -3.21 | 0.00** | -0.30 | [-0.48, -0.12] |
|  | Condition | 0.02 | 0.01 | 2.04 | 0.04* | 0.13 | [ 0.00, 0.25] |
|  | Street*Condition | -0.02 | 0.01 | -2.22 | 0.03* | -0.14 | [-0.26, -0.02] |
| *Note.* Sig. codes: 0 ‘***’ 0.001 ‘**’ 0.01 ‘*’ 0.05 ‘.’ 0.1 ‘ ’ 1 | | | | | | | |

To determine if the main effects of *Street* and *Condition* found with the LMM on participant’s broader visual attraction can be interpreted, we checked if the interaction was uncrossed (ordinal) or crossed (disordinal). As Figure 5B (reported in the main body of the manuscript) shows a disordinal interaction, the interpretation of the results will be focused on the interaction (Reinard, 2006). For the investigation of interaction, we run a descriptive analysis based on the percentage of overlap of the confidence intervals (CIs) as suggested by Garofalo et al. (2022). Indeed, Garofalo et al. (2022) demonstrated how post-hoc pairwise comparisons are often used and misused, and they put forward arguments in favour of analysing interactions effects via estimated marginal means and CIs. They argue that: “If there is little (< 25% of the full CI length) or no overlap, there is reasonable evidence of a difference between the two population means” (Garofalo et al., 2022, p.8). Estimated marginal means were calculated with the function “estimate_means” function of the “modelbased” R package (Makowski et al., 2020). The percentage of overlap between the intervals was calculated with the “perc_overlap” function of the “HTSSIP” R package (Youngblut et al., 2018). All CI, except for the overlaps involving the Green-Street/Art-Graetzloase CIs, share more than 25% percent of overlap between each other. Indeed, there is little overlap only between the Green-Street/Art-Graetzloase and Art-Street/Art-Graetzloase CIs (0%) and between the Green-Street/Art-Graetzloase and Art-Street/Green-Graetzloase CIs (0%). Consequently, there is reasonable evidence showing that the Art Graetzloase in the Green-Street was present in the participant visual field for longer times.

## Precise visual attraction

**Supplementary Table S6**

***Full report of the Precise Visual Attraction model’s result.***

|  |  | B | *SE* | *t* | *p* | *β* | 95% CI |
| --- | --- | --- | --- | --- | --- | --- | --- |
| Fixed effects | |  |  |  |  |  |  |
|  | Street | -0.09 | 0.23 | -0.41 | 0.68 | -0.09 | [-0.55, 0.36] |
|  | Condition | 0.54 | 0.10 | 5.56 | < .001*** | 0.54 | [ 0.35, 0.74] |
|  | Street*Condition | -0.27 | 0.10 | -2.82 | 0.00** | -0.27 | [-0.47, -0.08] |
| *Note*. Sig. codes: 0 ‘***’ 0.001 ‘**’ 0.01 ‘*’ 0.05 ‘.’ 0.1 ‘ ’ 1 | | | | | | | |

To determine if the main effect of *Condition* found with the GLMM on participant’s precise visual attraction can be interpreted, we checked if the interaction was ordinal or disordinal. Figure 5D shows an ordinal interaction, hence it was decided to investigate both the *Street*x*Condition* interaction and the effect of *Condition* (Reinard, 2006). To further interpret the main effect, the mean for the Art Graetzloases in Art-Street and Green-Street and the mean for the Green Graetzloases in Art-Street and Green-Street were computed. They indicate that participants looked for longer times at the Art Graetzloases (*M* = .02, *SD* = .03) than the Green one (*M* = .01, *SD* = .01). The Garofalo et al. (2022) method was employed to analyse the interaction. There is little overlap only between the Green-Street/Green-Graetzloase and Art-Street/Art-Graetzloase CIs (18.85%) and between the Art-Street/Green-Graetzloase and Green-Street/Art-Graetzloase CIs (13.23%). Consequently, there is reasonable evidence showing that the Green Graetzloase in the Green-Street was looked at less than the Art Graetzloase in the Art-Street. There is also reasonable evidence showing that the Green Graetzloase in the Art-Street was looked at less than the Art Graetzloase in the Green-Street. All the other CI shared more than 25% percent of overlap between each other, indicating no differences between the groups.

## Spatial attraction

**Supplementary Table S7**

***Full report of the Spatial Attraction model’s result.***

|  |  | B | *SE* | *t* | *p* | *β* | 95% CI |
| --- | --- | --- | --- | --- | --- | --- | --- |
| Fixed effect | |  |  |  |  |  |  |
|  | Condition | 0.16 | 0.11 | 1.45 | 0.15 | 0.16 | [-0.06, 0.37] |

# References

Garofalo, S., Giovagnoli, S., Orsoni, M., Starita, F., and Benassi, M. (2022). Interaction effect: Are you doing the right thing? PLoS ONE, 17(7), e0271668. doi: 10.1371/journal.pone.0271668

Holmqvist, K., Nyström, N., Andersson, R., Dewhurst, R., Jarodzka, H., & Van de Weijer, J. (Eds.) (2011). Eye tracking: a comprehensive guide to methods and measures, Oxford, UK: Oxford University Press.

Holmqvist, K. et al. (2021). Retracted article: Eye Tracking: Empirical Foundations for a minimal reporting guideline, Behavior Research Methods, 55(1), pp. 364–416. doi:10.3758/s13428-021-01762-8.

Landis, J. R., and Koch, G. G. (1977). The Measurement of Observer Agreement for Categorical Data. Biometrics, 33(1), 159. doi: 10.2307/2529310

Makowski, D., Ben-Shachar, M. S., Patil, I., and Lüdecke, D. (2020). Estimation of Model-Based Predictions, Contrasts and Means. CRAN. <https://github.com/easystats/modelbased>

Microsoft Corporation (2018). Microsoft Excel. Available at: https://office.microsoft.com/excel

Punde, P., Jadhav, M., and Manza, R. (2017). A study of eye tracking technology and its applications (p. 90). doi: 10.1109/ICISIM.2017.8122153

Pupil invisible - eye tracking glasses technical specifications (no date). Eye tracking technology - Gain insight into human behavior - Pupil Labs. https://pupil-labs.com/products/invisible/tech-specs [Accessed 26 February 2024].

Reinard, J. (2006). Communication Research Statistics, Sage Research Methods [Preprint]. doi:10.4135/9781412983693. [Accessed 26 February 2024].

Salvucci, D., and Goldberg, J. (2000). Identifying fixations and saccades in eye-tracking protocols. In Proceedings of the Eye Tracking Research and Applications Symposium (p. 78). doi: 10.1145/355017.355028

Technical overview (no date). Invisible - Technical Overview - Pupil Labs Docs. https://docs.pupil-labs.com/invisible/hardware/technical-overview/ [Accessed 26 February 2024].

Tobii Pro Glasses 3: Latest in wearable eye tracking (no date). Tobii. https://www.tobii.com/products/eye-trackers/wearables/tobii-pro-glasses-3#specifications [Accessed 26 February 2024].

Tobii Pro Glasses 3 User Manual (2024, v 1.19) Tobii. <https://go.tobii.com/tobii-pro-glasses-3-user-manual> [Accessed 26 February 2024].

Tonsen, M., Baumann, C. K., and Dierkes, K. (2020). A High-Level Description and Performance Evaluation of Pupil Invisible. doi: 10.48550/arXiv.2009.00508

Warrens, M. (2015). Five Ways to Look at Cohen’s Kappa. Journal of Psychology & Psychotherapy, 05. doi: 10.4172/2161-0487.1000197

Youngblut, N.D., Barnett, S.E., and Buckley, D.H. (2018). HTSSIP: An R package for analysis of high throughput sequencing data from nucleic acid stable isotope probing (SIP) experiments. PLoS ONE 13(1): e0189616. doi: 10.1371/journal.pone.0189616

1. Website URL: <https://www.tobii.com/products/eye-trackers/wearables/tobii-pro-glasses-3#specifications> [↑](#footnote-ref-1)
2. Website URL: <https://go.tobii.com/tobii-pro-glasses-3-user-manual> [↑](#footnote-ref-2)
3. Website URL: <https://pupil-labs.com/products/invisible/tech-specs> [↑](#footnote-ref-3)
4. Website URL: <https://docs.pupil-labs.com/invisible/hardware/technical-overview/> [↑](#footnote-ref-4)
